# Supplementary material for: Nuclear envelope-associated lipid droplets are enriched in cholesteryl esters and increase during inflammatory signaling
Source: EMBO J. 2025 Apr 7;44(10):2774–802. doi: 10.1038/s44318-025-00423-2 (PMC12084420; doi:10.1038/s44318-025-00423-2)
Supplement: Supplementary file 1 — Appendix [file 44318_2025_423_MOESM1_ESM.pdf]

Appendix for Nuclear-envelope-associated lipid droplets are enriched in cholesteryl esters and increase during inflammatory signaling

Ábel Szkalisity[1,2,*], Lauri Vanharanta[1,2,*], Hodaka Saito[1,2], Csaba Vörös[1,2,3], Shiqian Li[1,2], Antti Isomäki[4], Teemu Tomberg[5], Clare Strachan[5], Ilya Belevich[6], Eija Jokitalo[6], and Elina Ikonen[1,2]

[1]Department of Anatomy and Stem Cells and Metabolism Research Program, Faculty of Medicine, University of Helsinki, 00014 Helsinki, Finland

[2]Minerva Foundation Institute for Medical Research, 00290 Helsinki, Finland

[3]Synthetic and Systems Biology Unit, Biological Research Centre (BRC), Hungarian Research Network (HUN-REN), 6726 Szeged, Hungary

[4]Biomedicum Imaging Unit, Department of Anatomy, Faculty of Medicine, University of Helsinki, 00290 Helsinki, Finland

[5]Division of Pharmaceutical Chemistry and Technology, Faculty of Pharmacy, University of Helsinki, 00014 Helsinki, Finland

[6]Electron Microscopy Unit, Institute of Biotechnology, Helsinki Institute of Life Science, University of Helsinki, Helsinki, Finland

[*]these authors contributed equally

Contents

1 **Appendix Figures** **3**

Appendix Figure S1 3

Appendix Figure S2 4

Appendix Figure S3 5

Appendix Figure S4 6

2 **Appendix Tables** **7**

Appendix Table S1 7

3 **Appendix Supplementary Methods** **7**

3.1 cDNA of the SOAT-GFP 7

3.1.1 SOAT1.linker.sfGFP (in pEFIRES-P) 7

3.1.2 SOAT1.linker.sfGFP.KASH2 (in pEFIRES-P) 8

3.1.3 Tag-SUN2 (in pcDNA4-HisMax C) 8

3.2 HDR template of the SOAT-SNAPf 9

3.2.1 Left arm.linker.SNAPf.right arm 9

1 Appendix Figures

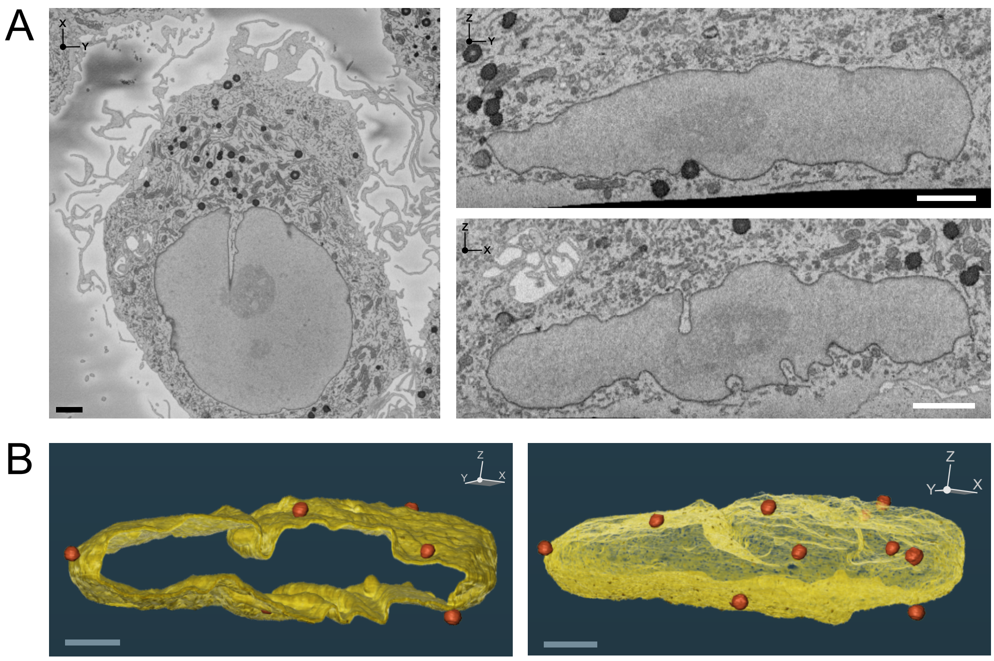

Appendix Figure S1: Nuclear invaginations and NE-LDs in control macrophages. (A) Orthogonal cross-sections taken across the nucleus from SBF-SEM data for a non-loaded control human primary macrophage. (B) Volume rendering of nuclear envelope (yellow) and model of the associated lipid droplets (vermilion) for the dataset represented in (A), similar layout to Figure 4B. Scale bar: 2 µm.

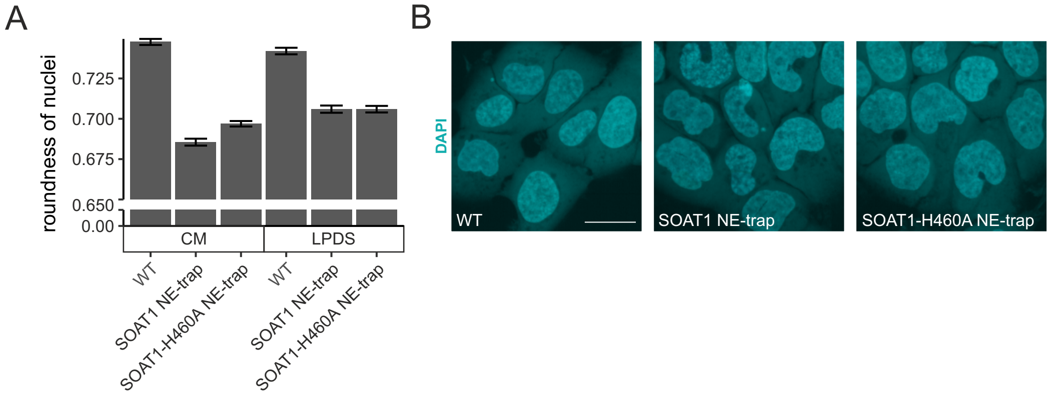

Appendix Figure S2: Roundness of nuclei in SOAT NE-trapped cells. (A) Mean and standard error of nuclear roundness ($4\pi *$area$/$perimeter^2) in wild-type, SOAT1 NE-trapped and SOAT1-H460A (inactive SOAT mutant) NE-trapped cells in complete medium or starvation in 5% LPDS for 3 days. Cell numbers: 3096, 3589, 4788, 2800, 2846, 3271 from left to right. (B) Exemplary images of DAPI staining for cells in complete medium. Scale-bar: 25 µm. It has previously been shown that SUN2 overexpression increases the irregularity of nuclear shape (*Krshnan et al. eLife 2022*, PMID: 36318477).

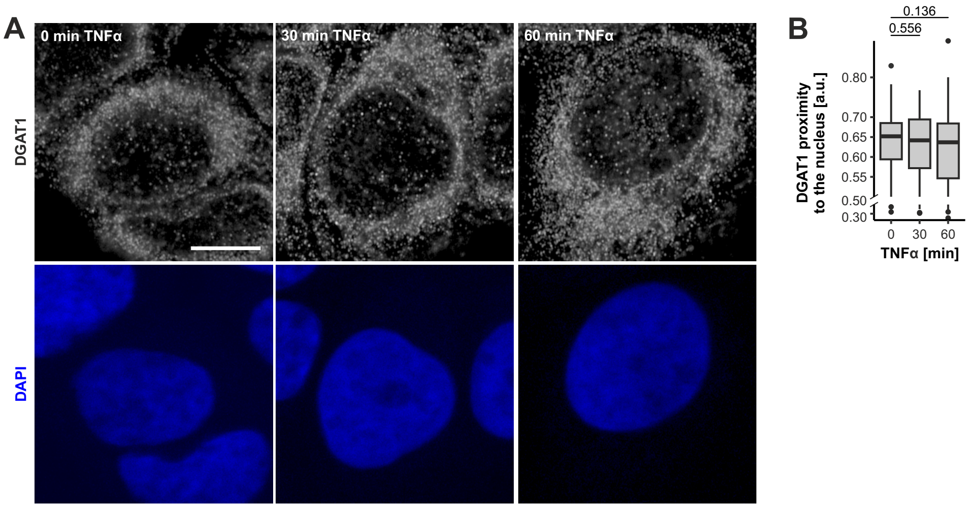

Appendix Figure S3: Localization of endogenous DGAT1 upon TNFα-treatment. (A) Exemplary images of A431 cells treated with TNFα, fixed and stained with DGAT1 antibody and DAPI. (B) Quantification of nuclear proximity of the DGAT1 signal. Cell numbers: 40, 61, 80 for 0, 30, 60 min TNFα load, p-values from *t*-tests. Scale-bar: 10 µm.

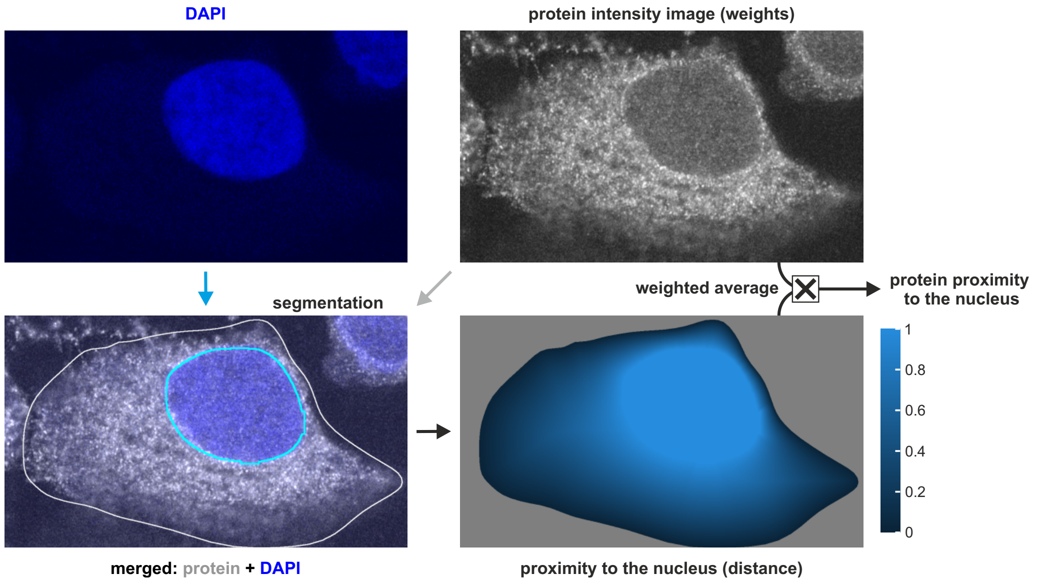

Appendix Figure S4: Definition of nuclear proximity. The nucleus and the plasma membrane are segmented based on the DAPI and protein channels (top row and bottom left). Based on the segmented masks for each pixel a normalized *nucleus - plasma membrane* distance is calculated as described in the methods. Briefly, the distance is a linearly interpolated value between 0 and 1: 1 is assigned to the nucleus and 0 to the plasma membrane (bottom right). This normalized distance image and protein intensity image is then combined with pixel-wise weighted averaging (the weights are the intensities, values are the distances) to form the *protein proximity to the nucleus* readout.

2 Appendix Tables

	Option 1		Option 2		Option 3	
Pump	Pump	Stokes	Pump	Stokes	Pump	Stokes
890 nm	15.6 mW<br>64.3 mJ/cm^2	44.7 mW<br>0.267 J/cm^2	25.0 mW<br>0.206 J/cm^2	63.6 mW<br>0.380 J/cm^2	41.3 mW<br>0.340 J/cm^2	94.6 mW<br>0.565 J/cm^2
805 nm	15.9 mW<br>0.160 J/cm^2	52.1 mW<br>0.311 J/cm^2	24.2 mW<br>0.244 J/cm^2	74.0 mW<br>0.442 J/cm^2	37.7 mW<br>0.380 J/cm^2	109.3 mW<br>0.653 J/cm^2
795 nm	16.3 mW<br>0.168 J/cm^2	52.4 mW<br>0.313 J/cm^2	24.5 mW<br>0.253 J/cm^2	74.4 mW<br>0.445 J/cm^2	38.0 mW<br>0.392 J/cm^2	109.5 mW<br>0.654 J/cm^2

Appendix Table S1: Laser power settings used for the SRS experiments depicted both as power (mW) and as fluence (J/cm^2), calculated based on the numerical aperture = 1.2, Gaussian approximation for beam radius 80 MHz repetition rate and 0.15 ps pulse duration.

3 Appendix Supplementary Methods

3.1 cDNA of the SOAT-GFP

3.1.1 SOAT1.linker.sfGFP (in pEFIRES-P)

ATGGTGGGTGAAGAGAAGATGTCTCTAAGAAACCGGCTGTCAAAGTCCAGGGAAAATCCTGAGGAAGATGAA
GACCAGAGAAACCCTGCAAAGGAGTCCCTAGAGACACCTAGTAATGGTCGAATTGACATAAAACAGTTGATA
GCAAAGAAGATAAAGTTGACAGCAGAGGCAGAGGAATTGAAGCCATTTTTTATGAAGGAAGTTGGCAGTCAC
TTTGATGATTTTGTGACCAATCTCATTGAAAAGTCAGCATCATTAGATAATGGTGGGTGCGCTCTCACAACCTT
TTCTGTTCTTGAAGGAGAGAAAAACAACCATAGAGCGAAGGATTTGAGAGCACCTCCAGAACAAGGAAAGAT
TTTTATTGCAAGGCGCTCTCTCTTAGATGAACTGCTTGAAGTGGACCACATCAGAACAATATATCACATGTTTA
TTGCCCTCCTCATTCTCTTTATCCTCAGCACACTTGTAGTAGATTACATTGATGAAGGAAGGCTGGTGCTTGAG
TTCAGCCTCCTGTCTTATGCTTTTGGCAAATTTCCTACCGTTGTTTGGACCTGGTGGATCATGTTCCTGTCTACA
TTTTCAGTTCCCTATTTTCTGTTTCAACATTGGGCCACTGGCTATAGCAAGAGTTCTCATCCGCTGATCCGTTCT
CTCTTCCATGGCTTTCTTTTCATGATCTTCCAGATTGGAGTTCTAGGTTTTGGACCAACATATGTTGTGTTAGCA
TATACACTGCCACCAGCTTCCCGGTTCATCATTATATTCGAGCAGATTCGTTTTGTAATGAAGGCCCACTCATT
TGTCAGAGAGAACGTGCCTCGGGTACTAAATTCAGCTAAGGAGAAATCAAGCACTGTTCCAATACCTACAGTC
AACCAGTATTTGTACTTCTTATTTGCTCCTACCCTTATCTACCGTGACAGCTATCCCAGGAATCCCACTGTAAGA
TGGGGTTATGTCGCTATGAAGTTTGCACAGGTCTTTGGTTGCTTTTTCTATGTGTACTACATCTTTGAAAGGCTT
TGTGCCCCCTTGTTTCGGAATATCAAACAGGAGCCCTTCAGCGCTCGTGTTCTGGTCCTATGTGTATTTAACTCC
ATCTTGCCAGGTGTGCTGATTCTCTTCCTTACTTTTTTTGCCTTTTTGCACTGCTGGCTCAATGCCTTTGCTGAGA
TGTTACGCTTTGGTGACAGGATGTTCTATAAGGATTGGTGGAACTCCACGTCATACTCCAACTATTATAGAACC
TGGAATGTGGTGGTCCATGACTGGCTATATTACTATGCTTACAAGGACTTTCTCTGGTTTTTCTCCAAGAGATTC
AAATCTGCTGCCATGTTAGCTGTCTTTGCTGTATCTGCTGTAGTACACGAATATGCCTTGGCTGTTTGCTTGAGC
TTTTTCTATCCCGTGCTCTTCGTGCTCTTCATGTTCTTTGGAATGGCTTTCAACTTCATTGTCAATGATAGTCGGA
AAAAGCCGATTTGGAATGTTCTGATGTGGACTTCTCTTTTCTTGGGCAATGGAGTCTTACTCTGCTTTTATTCTC
AAGAATGGTATGCACGTCAGCACTGTCCTCTGAAAAATCCCACATTTTTGGATTATGTCCGGCCACGTTCCTGG
ACTTGTCGTTACGTGTTT
GGATCTGGCGGAGGAGGGAGCGGGGGAGGCGGATCTGGCGGAGGCGGATCCCTCGAG
ATGGTGAGCAAGGGCGAGGAGCTGTTCACCGGGGTGGTGCCCATCCTGGTCGAGCTGGACGGCGACGTAAAC
GGCCACAAGTTCAGCGTGCGCGGCGAGGGCGAGGGCGATGCCACCAACGGCAAGCTGACCCTGAAGTTCATC
TGCACCACCGGCAAGCTGCCCGTGCCCTGGCCCACCCTCGTGACCACCCTGACCTACGGCGTGCAGTGCTTCA
GCCGCTACCCCGACCACATGAAGCGCCACGACTTCTTCAAGTCCGCCATGCCCGAAGGCTACGTCCAGGAGCG
CACCATCAGCTTCAAGGACGACGGCACCTACAAGACCCGCGCCGAGGTGAAGTTCGAGGGCGACACCCTGGT
GAACCGCATCGAGCTGAAGGGCATCGACTTCAAGGAGGACGGCAACATCCTGGGGCACAAGCTGGAGTACAA
CTTCAACAGCCACAACGTCTATATCACCGCCGACAAGCAGAAGAACGGCATCAAGGCCAACTTCAAGATCCGC
CACAACGTGGAGGACGGCAGCGTGCAGCTCGCCGACCACTACCAGCAGAACACCCCCATCGGCGACGGCCCC
GTGCTGCTGCCCGACAACCACTACCTGAGCACCCAGTCCGTGCTGAGCAAAGACCCCAACGAGAAGCGCGATC
ACATGGTCCTGCTGGAGTTCGTGACCGCCGCCGGGATCACTCACGGCATGGACGAGCTGTACAAGTAA

3.1.2 SOAT1.linker.sfGFP.KASH2 (in pEFIRES-P)

ATGGTGGGTGAAGAGAAGATGTCTCTAAGAAACCGGCTGTCAAAGTCCAGGGAAAATCCTGAGGAAGATGAA
GACCAGAGAAACCCTGCAAAGGAGTCCCTAGAGACACCTAGTAATGGTCGAATTGACATAAAACAGTTGATA
GCAAAGAAGATAAAGTTGACAGCAGAGGCAGAGGAATTGAAGCCATTTTTTATGAAGGAAGTTGGCAGTCAC
TTTGATGATTTTGTGACCAATCTCATTGAAAAGTCAGCATCATTAGATAATGGTGGGTGCGCTCTCACAACCTT
TTCTGTTCTTGAAGGAGAGAAAAACAACCATAGAGCGAAGGATTTGAGAGCACCTCCAGAACAAGGAAAGAT
TTTTATTGCAAGGCGCTCTCTCTTAGATGAACTGCTTGAAGTGGACCACATCAGAACAATATATCACATGTTTA
TTGCCCTCCTCATTCTCTTTATCCTCAGCACACTTGTAGTAGATTACATTGATGAAGGAAGGCTGGTGCTTGAG
TTCAGCCTCCTGTCTTATGCTTTTGGCAAATTTCCTACCGTTGTTTGGACCTGGTGGATCATGTTCCTGTCTACA
TTTTCAGTTCCCTATTTTCTGTTTCAACATTGGGCCACTGGCTATAGCAAGAGTTCTCATCCGCTGATCCGTTCT
CTCTTCCATGGCTTTCTTTTCATGATCTTCCAGATTGGAGTTCTAGGTTTTGGACCAACATATGTTGTGTTAGCA
TATACACTGCCACCAGCTTCCCGGTTCATCATTATATTCGAGCAGATTCGTTTTGTAATGAAGGCCCACTCATT
TGTCAGAGAGAACGTGCCTCGGGTACTAAATTCAGCTAAGGAGAAATCAAGCACTGTTCCAATACCTACAGTC
AACCAGTATTTGTACTTCTTATTTGCTCCTACCCTTATCTACCGTGACAGCTATCCCAGGAATCCCACTGTAAGA
TGGGGTTATGTCGCTATGAAGTTTGCACAGGTCTTTGGTTGCTTTTTCTATGTGTACTACATCTTTGAAAGGCTT
TGTGCCCCCTTGTTTCGGAATATCAAACAGGAGCCCTTCAGCGCTCGTGTTCTGGTCCTATGTGTATTTAACTCC
ATCTTGCCAGGTGTGCTGATTCTCTTCCTTACTTTTTTTGCCTTTTTGCACTGCTGGCTCAATGCCTTTGCTGAGA
TGTTACGCTTTGGTGACAGGATGTTCTATAAGGATTGGTGGAACTCCACGTCATACTCCAACTATTATAGAACC
TGGAATGTGGTGGTCCATGACTGGCTATATTACTATGCTTACAAGGACTTTCTCTGGTTTTTCTCCAAGAGATTC
AAATCTGCTGCCATGTTAGCTGTCTTTGCTGTATCTGCTGTAGTACACGAATATGCCTTGGCTGTTTGCTTGAGC
TTTTTCTATCCCGTGCTCTTCGTGCTCTTCATGTTCTTTGGAATGGCTTTCAACTTCATTGTCAATGATAGTCGGA
AAAAGCCGATTTGGAATGTTCTGATGTGGACTTCTCTTTTCTTGGGCAATGGAGTCTTACTCTGCTTTTATTCTC
AAGAATGGTATGCACGTCAGCACTGTCCTCTGAAAAATCCCACATTTTTGGATTATGTCCGGCCACGTTCCTGG
ACTTGTCGTTACGTGTTT
GGATCTGGCGGAGGAGGGAGCGGGGGAGGCGGATCTGGCGGAGGCGGATCCCTCGAG
ATGGTGAGCAAGGGCGAGGAGCTGTTCACCGGGGTGGTGCCCATCCTGGTCGAGCTGGACGGCGACGTAAAC
GGCCACAAGTTCAGCGTGCGCGGCGAGGGCGAGGGCGATGCCACCAACGGCAAGCTGACCCTGAAGTTCATC
TGCACCACCGGCAAGCTGCCCGTGCCCTGGCCCACCCTCGTGACCACCCTGACCTACGGCGTGCAGTGCTTCA
GCCGCTACCCCGACCACATGAAGCGCCACGACTTCTTCAAGTCCGCCATGCCCGAAGGCTACGTCCAGGAGCG
CACCATCAGCTTCAAGGACGACGGCACCTACAAGACCCGCGCCGAGGTGAAGTTCGAGGGCGACACCCTGGT
GAACCGCATCGAGCTGAAGGGCATCGACTTCAAGGAGGACGGCAACATCCTGGGGCACAAGCTGGAGTACAA
CTTCAACAGCCACAACGTCTATATCACCGCCGACAAGCAGAAGAACGGCATCAAGGCCAACTTCAAGATCCGC
CACAACGTGGAGGACGGCAGCGTGCAGCTCGCCGACCACTACCAGCAGAACACCCCCATCGGCGACGGCCCC
GTGCTGCTGCCCGACAACCACTACCTGAGCACCCAGTCCGTGCTGAGCAAAGACCCCAACGAGAAGCGCGATC
ACATGGTCCTGCTGGAGTTCGTGACCGCCGCCGGGATCACTCACGGCATGGACGAGCTGTACAAG
GGATCC
CCCTCCTCCGAAGAAGACTACAGCTGCACTCAGGCCAACAACTTTGCCCGGTCCTTTTACCCCATGCTGAGGTA
CACCAATGGGCCACCCCCCACATAG

3.1.3 Tag-SUN2 (in pcDNA4-HisMax C)

ATGGGGGGTTCTCATCATCATCATCATCATGGTATGGCTAGCATGACTGGTGGACAGCAAATGGGTCGGGATC
TGTACGACGATGACGATAAGGTACCAGGATCTACC
ATGTCCCGAAGAAGCCAGCGCCTCACGCGCTACTCCCAGGGTGACGATGACGGCAGCAGCAGCAGCGGAGGG
AGCTCGGTGGCTGGGAGTCAGAGCACCCTGTTTAAAGACAGTCCTCTCAGGACCTTGAAGAGGAAATCCAGCA
ACATGAAGCGCCTGTCCCCAGCGCCACAGCTGGGCCCGTCCTCTGATGCACACACCTCCTACTACAGTGAGTC
GCTGGTCCACGAGTCCTGGTTCCCACCCAGGAGCTCCCTGGAGGAACTGCATGGTGACGCCAACTGGGGTGAG
GACCTGCGGGTGCGGAGGAGGAGAGGCACGGGTGGCTCAGAGAGCAGCAGGGCCAGCGGGCTTGTGGGGCGC
AAGGCCACCGAGGACTTCCTGGGCTCTTCCTCGGGCTACTCCTCTGAGGACGACTACGTGGGCTACTCGGATGT
GGACCAGCAGAGTTCCAGCTCGCGGCTCCGAAGCGCCGTCTCACGGGCGGGCTCCTTACTCTGGATGGTGGCC
ACTTCGCCAGGCCGGCTCTTCAGACTTCTCTACTGGTGGGCTGGCACCACCTGGTACCGCCTGACCACAGCTGC
CTCCCTCCTTGACGTCTTCGTTTTAACCAGGCGCTTCTCGTCCCTGAAGACGTTCCTCTGGTTCCTGCTGCCGCT
GCTCTTGCTGACGTGCCTGACGTATGGTGCTTGGTATTTCTACCCCTATGGGCTGCAGACATTCCACCCTGCTTT
GGTTTCCTGGTGGGCAGCGAAGGACAGCAGGAGGCCGGATGAGGGCTGGGAAGCCAGAGACTCATCGCCACA
TTTCCAGGCTGAGCAGCGTGTTATGTCCCGGGTACACTCTCTGGAGCGGCGTCTGGAAGCTCTTGCTGCTGAAT
TTTCCTCCAACTGGCAGAAGGAGGCCATGCGGCTGGAACGTCTGGAGCTGCGGCAAGGGGCTCCTGGCCAGGG
AGGTGGTGGTGGCCTGAGCCACGAGGACACCCTGGCGCTGCTGGAGGGGCTAGTGAGCCGCCGTGAAGCTGC
CCTGAAGGAGGATTTCCGCAGGGAAACTGCTGCTCGCATCCAGGAAGAACTGTCTGCCCTGAGAGCAGAGCAT
CAGCAAGACTCAGAAGACCTCTTCAAGAAGATCGTCCGGGCCTCCCAGGAGTCCGAGGCTCGCATCCAGCAGC
TGAAGTCAGAGTGGCAAAGCATGACCCAGGAGTCCTTCCAGGAGAGCTCTGTGAAGGAGCTGAGGCGGCTGG
AGGACCAGCTGGCCGGCCTGCAGCAGGAGCTGGCGGCTCTGGCACTGAAGCAGAGCTCGGTGGCGGAAGAAG
TGGGCCTGCTGCCCCAGCAGATCCAGGCCGTGCGGGACGACGTGGAATCTCAGTTCCCGGCCTGGATCAGTCA
GTTCCTTGCCCGAGGTGGAGGGGGCCGCGTGGGGCTCCTTCAGAGAGAGGAGATGCAAGCTCAGCTGCGAGA

GCTGGAGAGCAAGATCCTCACCCATGTGGCAGAGATGCAGGGCAAGTCGGCCAGGGAAGCCGCGGCCTCCCT
GAGCCTGACGCTGCAGAAAGAAGGTGTGATTGGAGTGACAGAGGAGCAGGTGCACCACATCGTGAAGCAGGC
CCTGCAGCGCTACAGTGAGGACCGCATCGGGCTGGCAGACTACGCCCTGGAGTCAGGAGGGGCCAGCGTCAT
CAGCACCCGATGTTCTGAGACCTACGAGACCAAGACGGCCCTCCTCAGCCTCTTCGGCATCCCCCTGTGGTACC
ACTCCCAGTCACCCCGAGTCATCCTCCAGCCAGATGTGCACCCAGGCAACTGCTGGGCCTTCCAGGGGCCACA
AGGCTTCGCCGTGGTCCGCCTCTCTGCCCGCATCCGCCCCACAGCCGTTACCTTAGAGCATGTGCCCAAGGCCT
TGTCACCCAACAGCACTATCTCCAGTGCCCCCAAGGACTTCGCCATCTTTGGGTTTGACGAAGACCTGCAGCAG
GAGGGGACACTCCTTGGCAAGTTCACTTACGATCAGGACGGCGAGCCTATTCAGACGTTTCACTTTCAGGCCC
CTACGATGGCCACGTACCAGGTGGTGGAGCTGCGGATCCTGACTAACTGGGGCCACCCCGAGTACACCTGCAT
CTACCGCTTCAGAGTGCATGGGGAGCCCGCCCACTAG

3.2 HDR template of the SOAT-SNAPf

3.2.1 Left arm.linker.SNAPf.right arm

GGTACTAAAAGTAGGGCTGTGCTAAAATAAAGATACAGAAAGAAATAGTGCCTATTTAGAAACTGGTCAAAG
TCCACACAATTGTGTATAGTTTACTGGACTTTAAAAGCTTTATGTGAAGCACCTAATTGAATAAATCTGTGTTA
TAGATAAAAGTACTGAACATCTTTTGAATTAGTGAGCTGCTTATACCTTGAGGGTTGTTGAAATGGTGGGAGGC
ATTTTAGAGATGTACAATGTATCATATTAACATCCCTGCCTATCTCCACACCTCCTCTACTCTGTACACAAATTA
TTTTTCCATTCTTATTTTTCCCCACTGCAGCCCACATTTTTGGATTATGTCCGGCCACGTTCCTGGACTTGTCGTT
ACGTGTTT GGATCTGGCGGAGGAGGGAGCGGGGGAGGCGGATCTGGCGGAGGCGGATCC ATGGACAAAGAC
TGCGAAATGAAGCGCACCACCCTGGATAGCCCTCTGGGCAAGCTGGAACTGTCTGGGTGCGAACAGGGCCTGC
ACCGTATCATCTTCCTGGGCAAAGGAACATCTGCCGCCGACGCCGTGGAAGTGCCTGCCCCAGCCGCCGTGCT
GGGCGGACCAGAGCCACTGATGCAGGCCACCGCCTGGCTCAACGCCTACTTTCACCAGCCTGAGGCCATCGAG
GAGTTCCCTGTGCCAGCCCTGCACCACCCAGTGTTCCAGCAGGAGAGCTTTACCCGCCAGGTGCTGTGGAAAC
TGCTGAAAGTGGTGAAGTTCGGAGAGGTCATCAGCTACAGCCACCTGGCCGCCCTGGCCGGCAATCCCGCCGC
CACCGCCGCCGTGAAAACCGCCCTGAGCGGAAATCCCGTGCCCATTCTGATCCCCTGCCACCGGGTGGTGCAG
GGCGACCTGGACGTGGGGGGCTACGAGGGCGGGCTCGCCGTGAAAGAGTGGCTGCTGGCCCACGAGGGCCAC
AGACTGGGCAAGCCTGGGCTGGGTTAG TGTACAAG TAGAAGCTTGGACTTTGTTTCCTCCTTGTCACTGAAGA
TTGGGTAGCTCCCTGATTTGGAGCCAGCTGTTTCCAGTTGTTACTGAAGTTATCTGTGTTATTTGGACCACTCCA
GGCTTTACAGATGACTCACTCCATTCCTAGGTCACTTGAAGCCAAACTGTTGGAAGTTCACTGGAGTCTTGTAC
ACTTAAGCAGAGCAGAACTTTTTTTGTGGGGCTGGGTGGGGGGAGAAGACCGACTAACAGCTGAAGTAATGAC
AGATTGTTGCTGGGTCATATCAGCTTTATCCCTTGGTAATTATATCTGTTTTGTTTCTTGACTCTGTCCAATCAG
AGAATAAACATCATAGTTTCTTGGCCACTGAATTAGCCAAAACACTTAGGAAGAAATCACTTAAATACCTCTG
GCTTAGAAATTTTTTCATGCACACTGTTGGAATGTATGCTAATTGAACATGCAATTGGGGAAGAAAAAATGTA
GAATGATTTTTGCTATTTCTAGTAGAAAGAAAATGTCTGTTTTCCAAAGATAATGTTATACATCCTATTTTGTAA
TTTTTTTGAAAAAAGTTCAATGTTCAGTTTTCCTTAGTTTTTACCTTGTTTTCTCTATAGGTCATGATTTCTGTGA
AGCAAAAAGATGCCTTTTACCATGAATTCTTGAGTTTACATCAATAATATTGTATATTAAGGGGATCAGAAGTA
GGAAGGAAAAAATAAGAGATAGCAGAGGAAAAAGAAAAACATTTCCTCTTATAACTTCTGAAGTAATTTGTA
AAAAAGATTTGTAGAGTCAATCATGTGTTTAAATTATTTTATCACAAACTTAACATGGAAGATATTCCTTTTTA
ACTTTGTGGTAACTTCTTTGAAGTTATTTAGAAATATCCTTTGGAACAATTATTTTATTGTCTAATAAATATTGA
CTTCTCTTGAATTATTTTGCAGACTAGTGAGTCTGTACCATAAGTATTAATCACCTCCACTCATATTAAAGTGAT
CATTAAGAATCCAGAAGCTGGCTTCTGCATTTGCTCAGTTATACTTTTAATGGTAGTATGTTTTTAGGTGGAAT
AAATTAATATGTGATTGGTTTCAAGGAAATGTACTCTATTATGTAATACTTCCATTTTATAAGATGCCCATTTCT
AATACAATGTGTGTAGGAATTATTTGTATGTATGAGGTATGATTGTAAAGATTGAGCATTGGAAGGGGTATCA
GAGACCATGTAGTTCAACTTTCCACTCAAAGTAAGATTTATGAATTATTTAAATGATAGTTGTTACTTGGAACA
GCCACTTGAGAGGCT
