# Supplementary figures and images for: Nuclear envelope-associated lipid droplets are enriched in cholesteryl esters and increase during inflammatory signaling
Source: EMBO J. 2025 Apr 7;44(10):2774–802. doi: 10.1038/s44318-025-00423-2 (PMC12084420; doi:10.1038/s44318-025-00423-2)

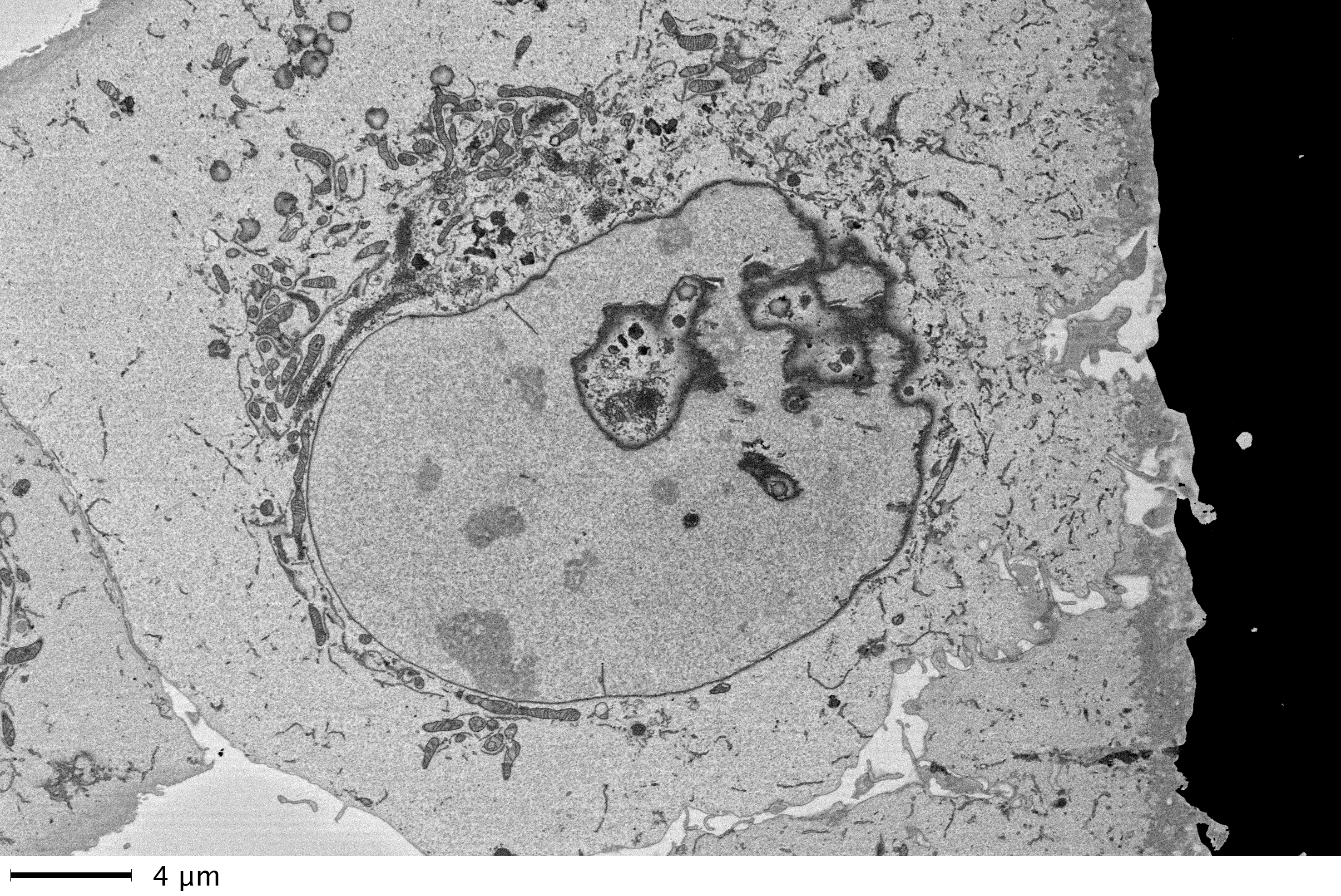

Supplement: Supplementary file 5 — Source data Fig. 3 [file 44318_2025_423_MOESM5_ESM.zip › 3C/A431_CM_SBF-SEM_bottom.tif]

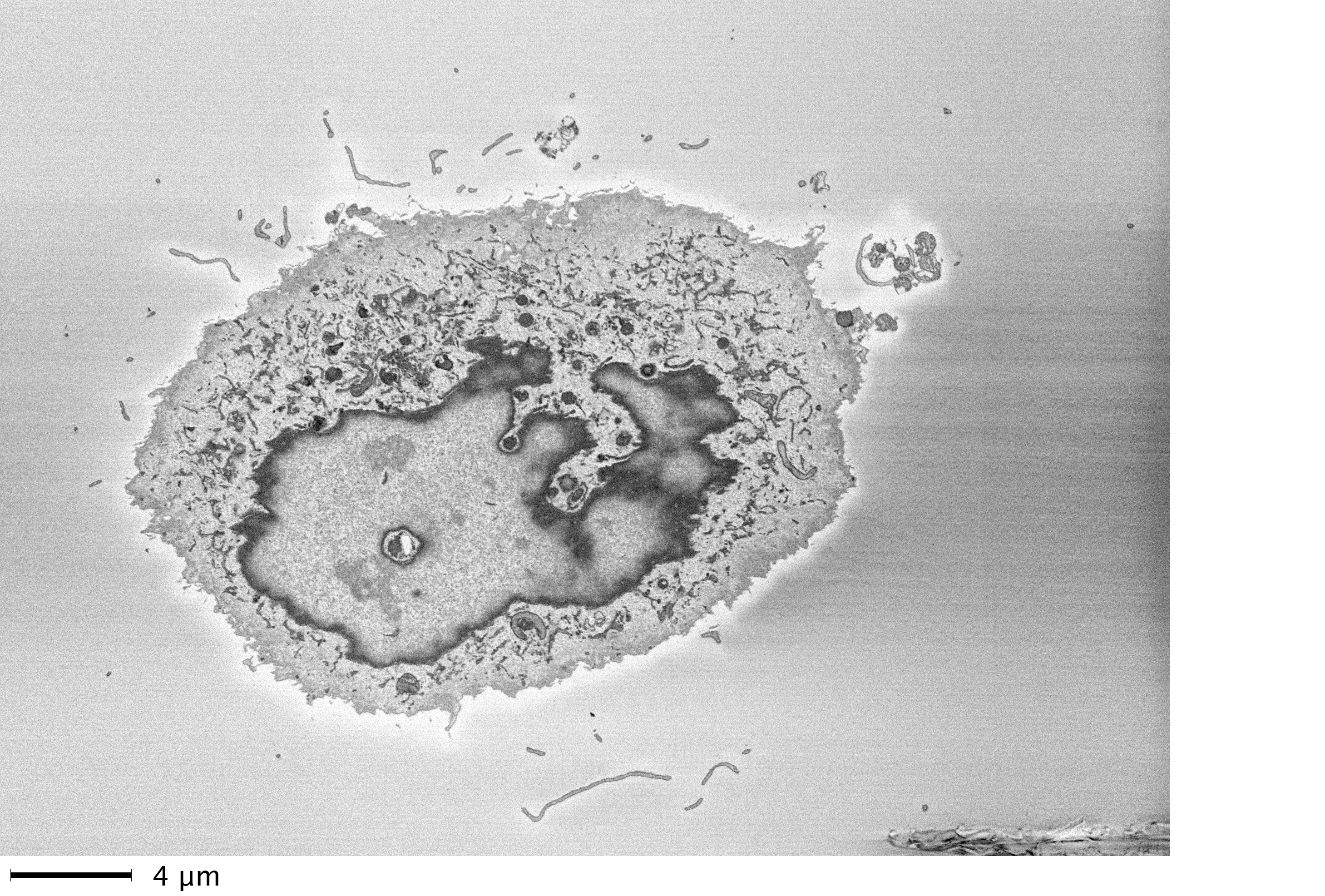

Supplement: Supplementary file 5 — Source data Fig. 3 [file 44318_2025_423_MOESM5_ESM.zip › 3C/A431_CM_SBF-SEM_top.tif]

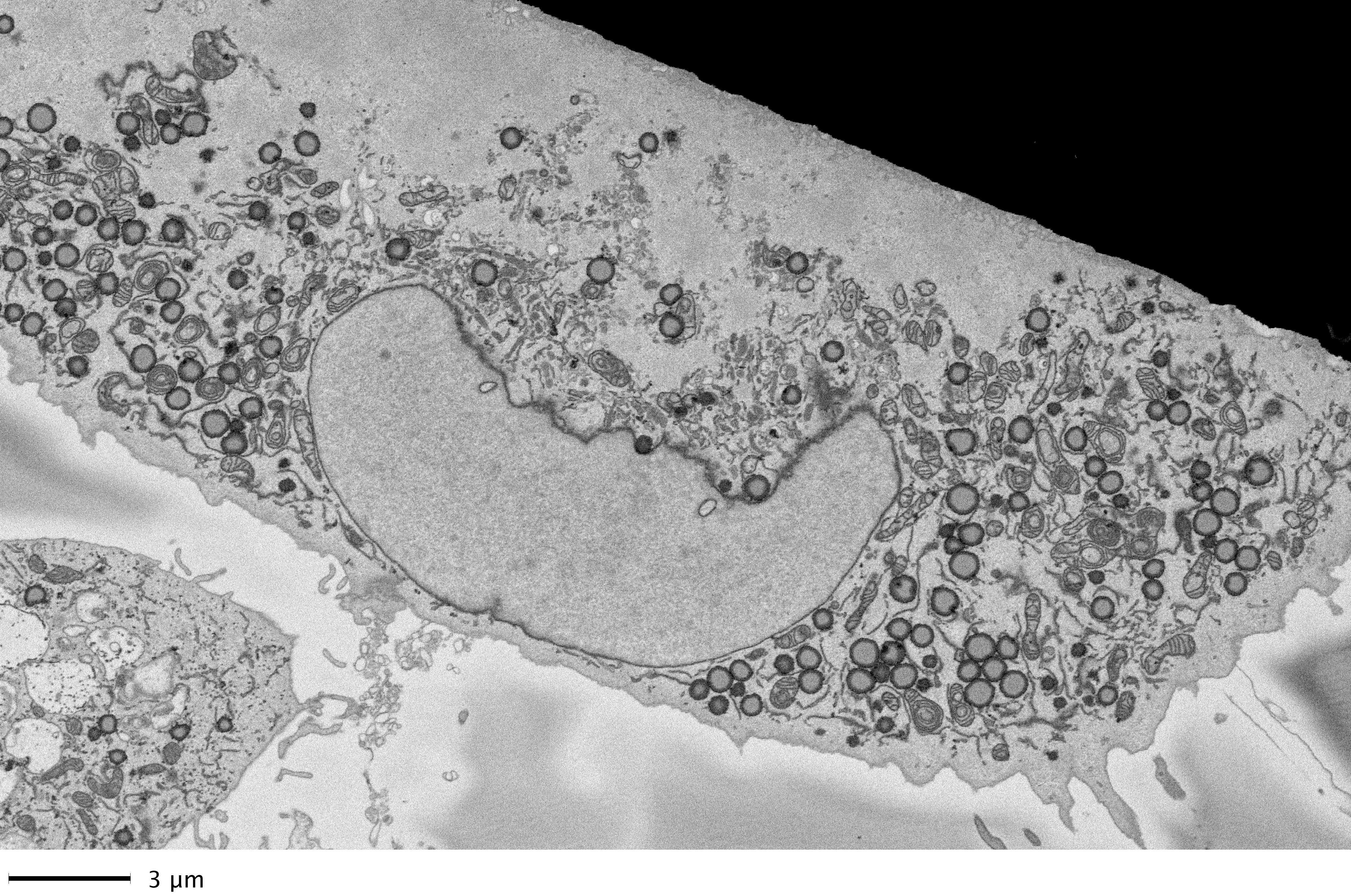

Supplement: Supplementary file 6 — Source data Fig. 4 [file 44318_2025_423_MOESM6_ESM.zip › 4A/Macrophage_2hChol+OA_XY.tif]

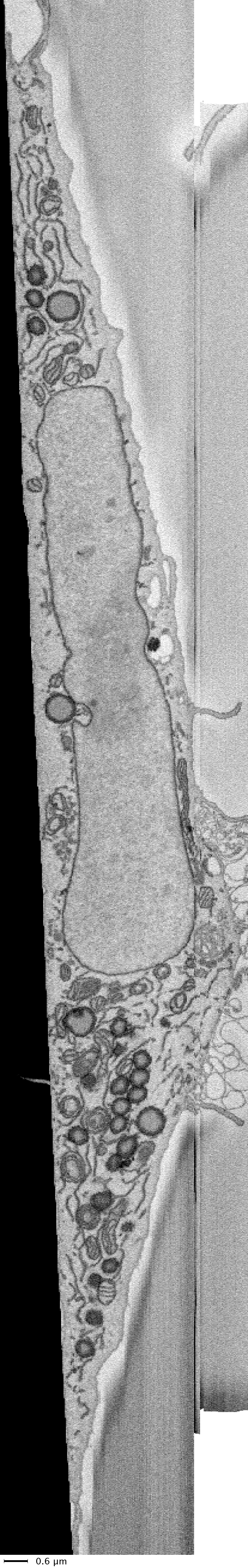

Supplement: Supplementary file 6 — Source data Fig. 4 [file 44318_2025_423_MOESM6_ESM.zip › 4A/Macrophage_2hChol+OA_ZX.tif]

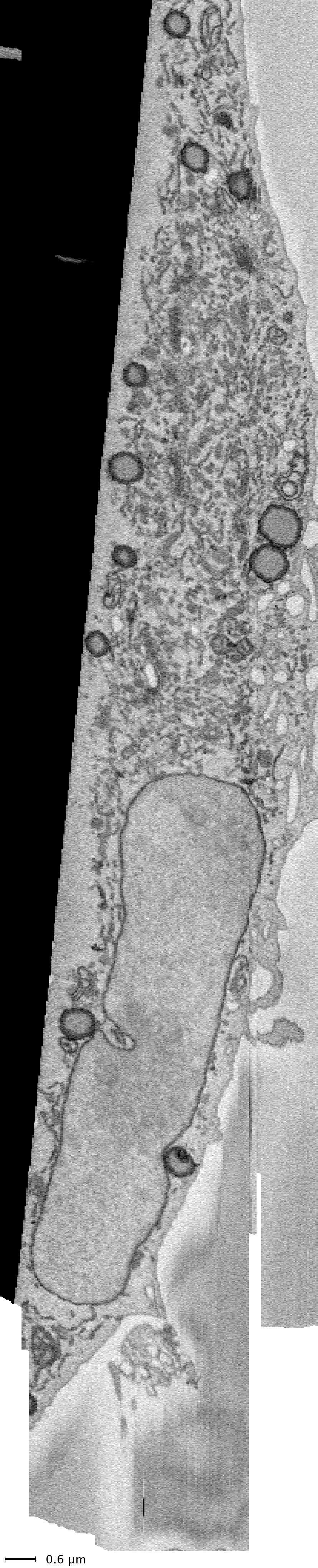

Supplement: Supplementary file 6 — Source data Fig. 4 [file 44318_2025_423_MOESM6_ESM.zip › 4A/Macrophage_2hChol+OA_ZY.tif]

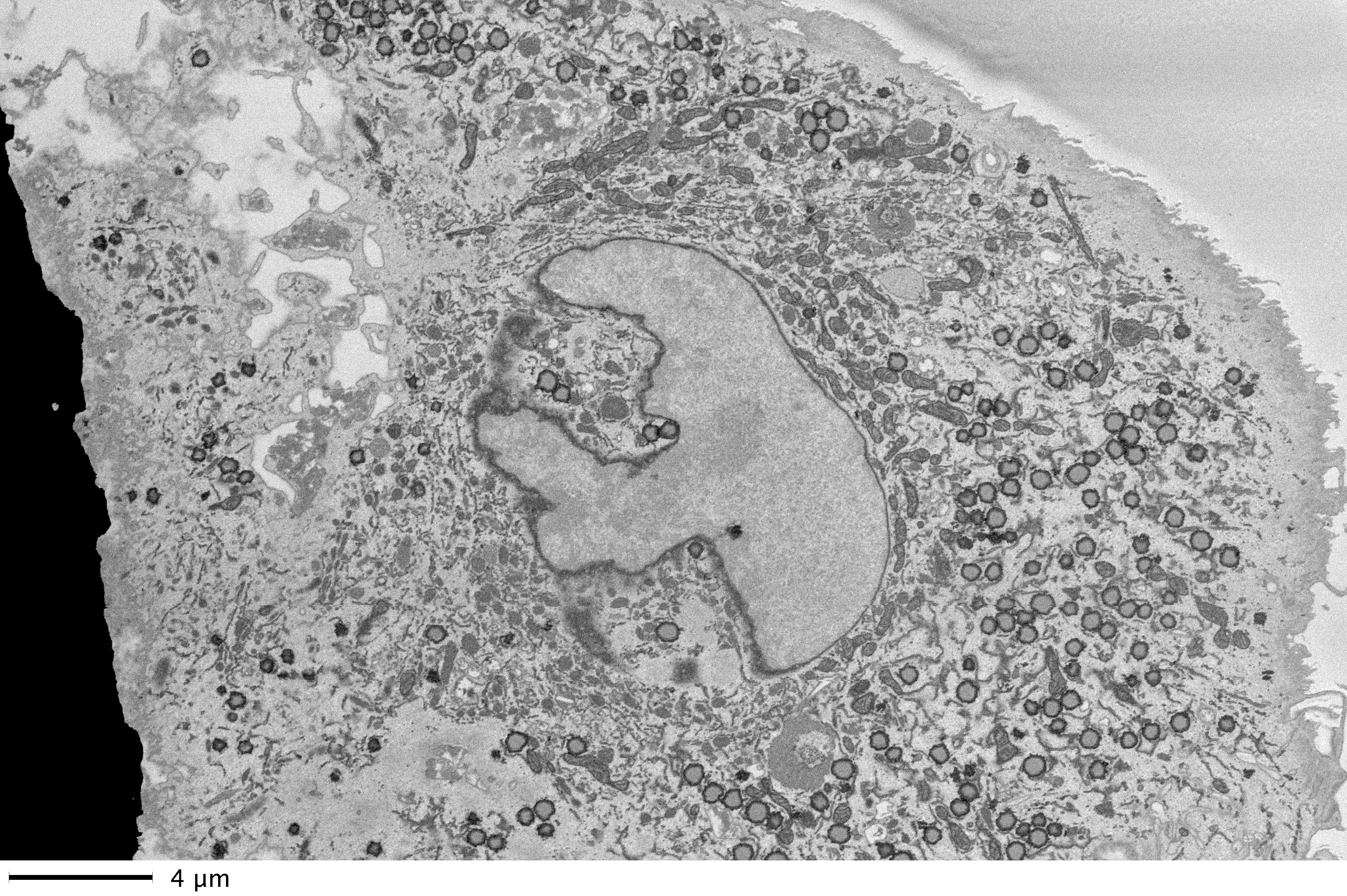

Supplement: Supplementary file 6 — Source data Fig. 4 [file 44318_2025_423_MOESM6_ESM.zip › 4C/Macrophage_2hChol+OA_XY.tif]

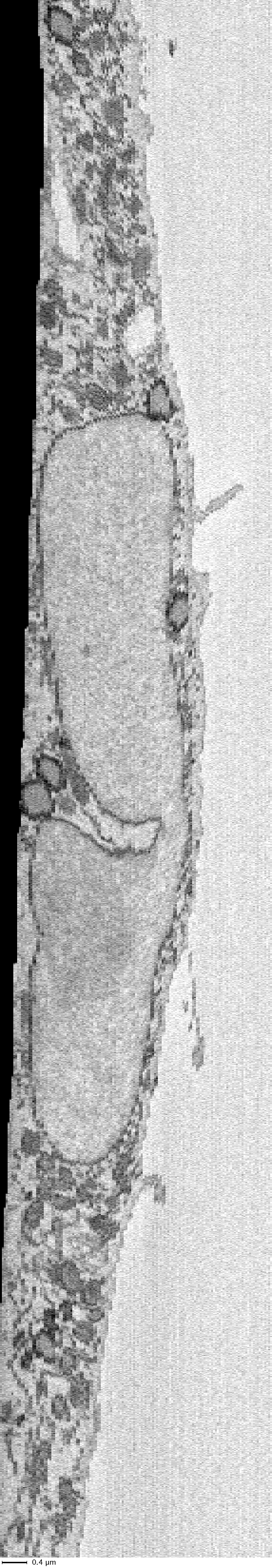

Supplement: Supplementary file 6 — Source data Fig. 4 [file 44318_2025_423_MOESM6_ESM.zip › 4C/Macrophage_2hChol+OA_ZX.tif]

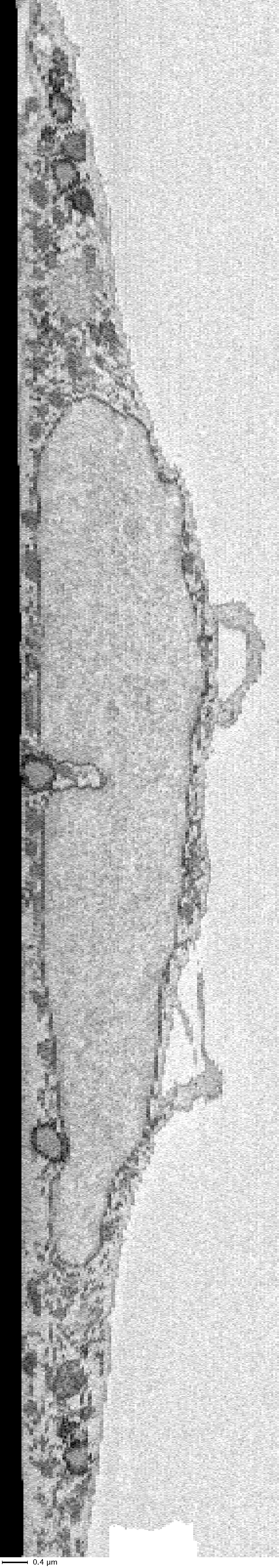

Supplement: Supplementary file 6 — Source data Fig. 4 [file 44318_2025_423_MOESM6_ESM.zip › 4C/Macrophage_2hChol+OA_ZY.tif]

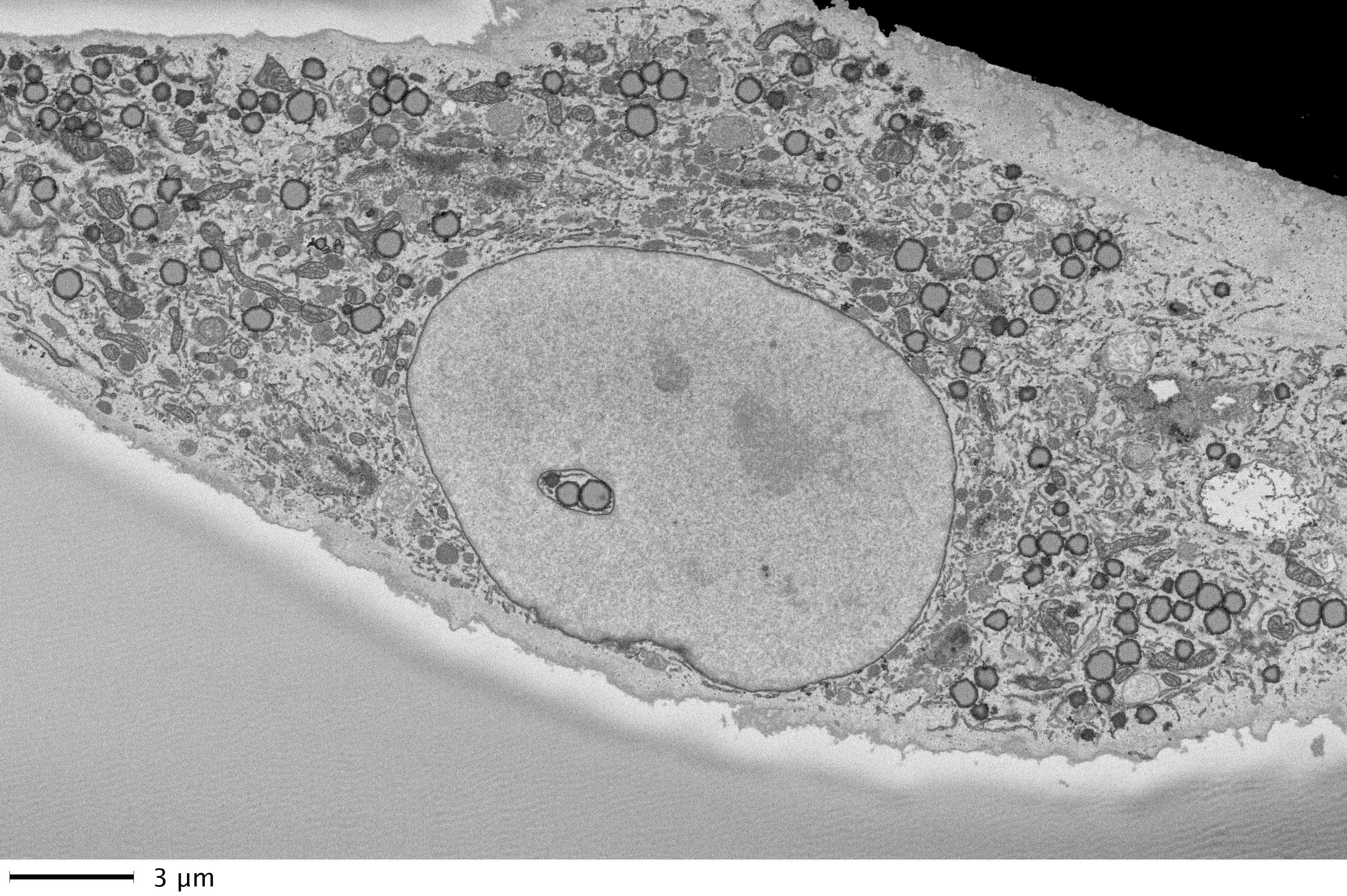

Supplement: Supplementary file 6 — Source data Fig. 4 [file 44318_2025_423_MOESM6_ESM.zip › 4E/Macrophage_2hChol+OA_XY.tif]

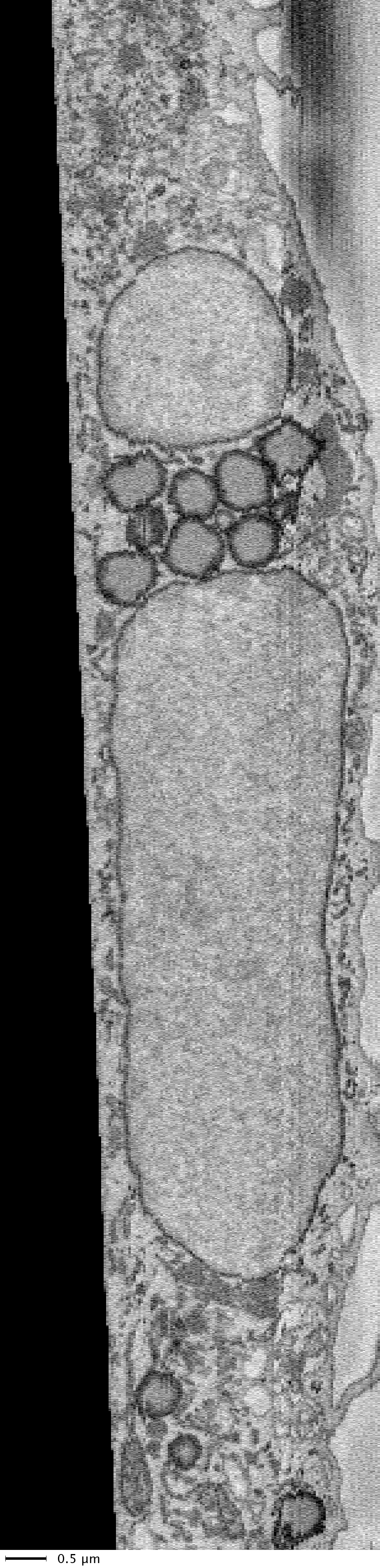

Supplement: Supplementary file 6 — Source data Fig. 4 [file 44318_2025_423_MOESM6_ESM.zip › 4E/Macrophage_2hChol+OA_ZX.tif]

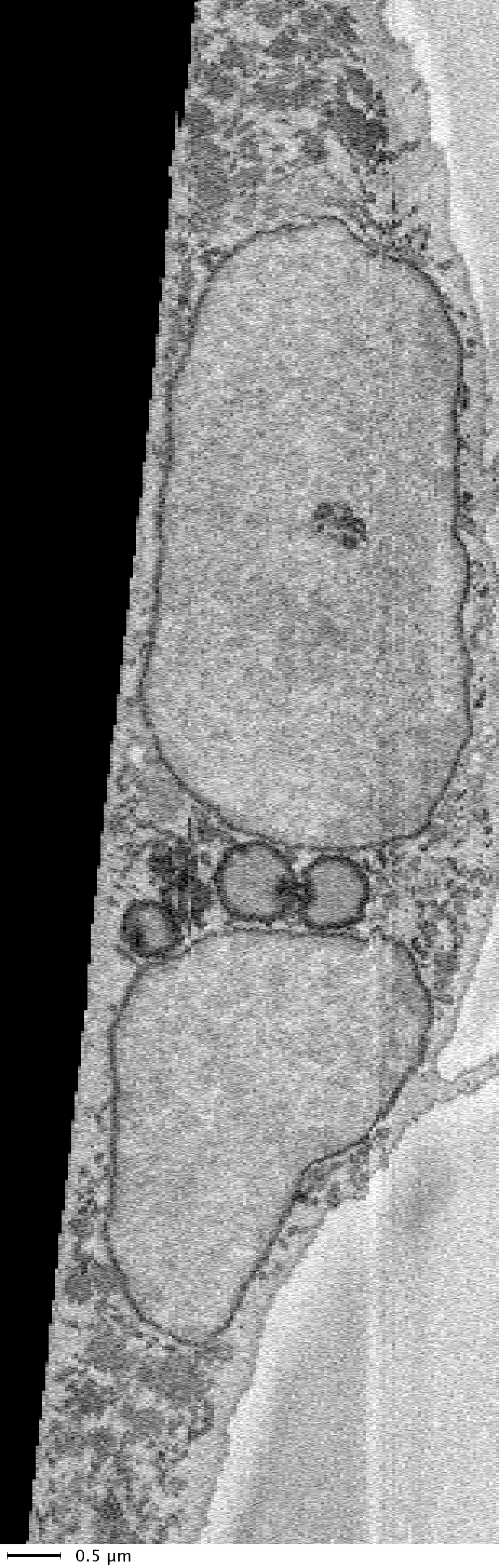

Supplement: Supplementary file 6 — Source data Fig. 4 [file 44318_2025_423_MOESM6_ESM.zip › 4E/Macrophage_2hChol+OA_ZY.tif]

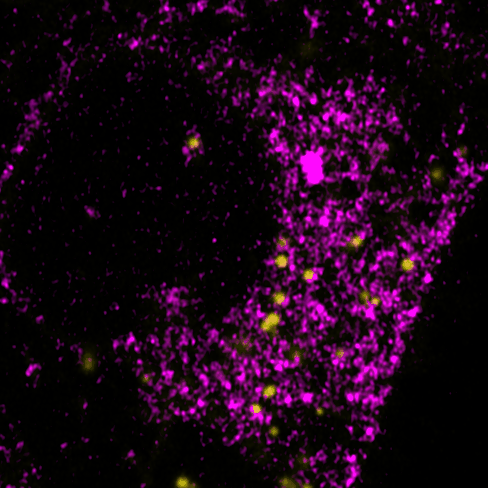

Supplement: Supplementary file 8 — Source data Fig. 6 [file 44318_2025_423_MOESM8_ESM.zip › 6G/A431_SOAT-SNAP_Live_Left.gif]

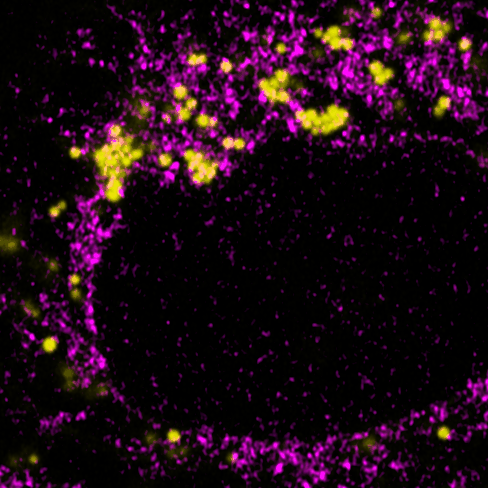

Supplement: Supplementary file 8 — Source data Fig. 6 [file 44318_2025_423_MOESM8_ESM.zip › 6G/A431_SOAT-SNAP_Live_Right.gif]

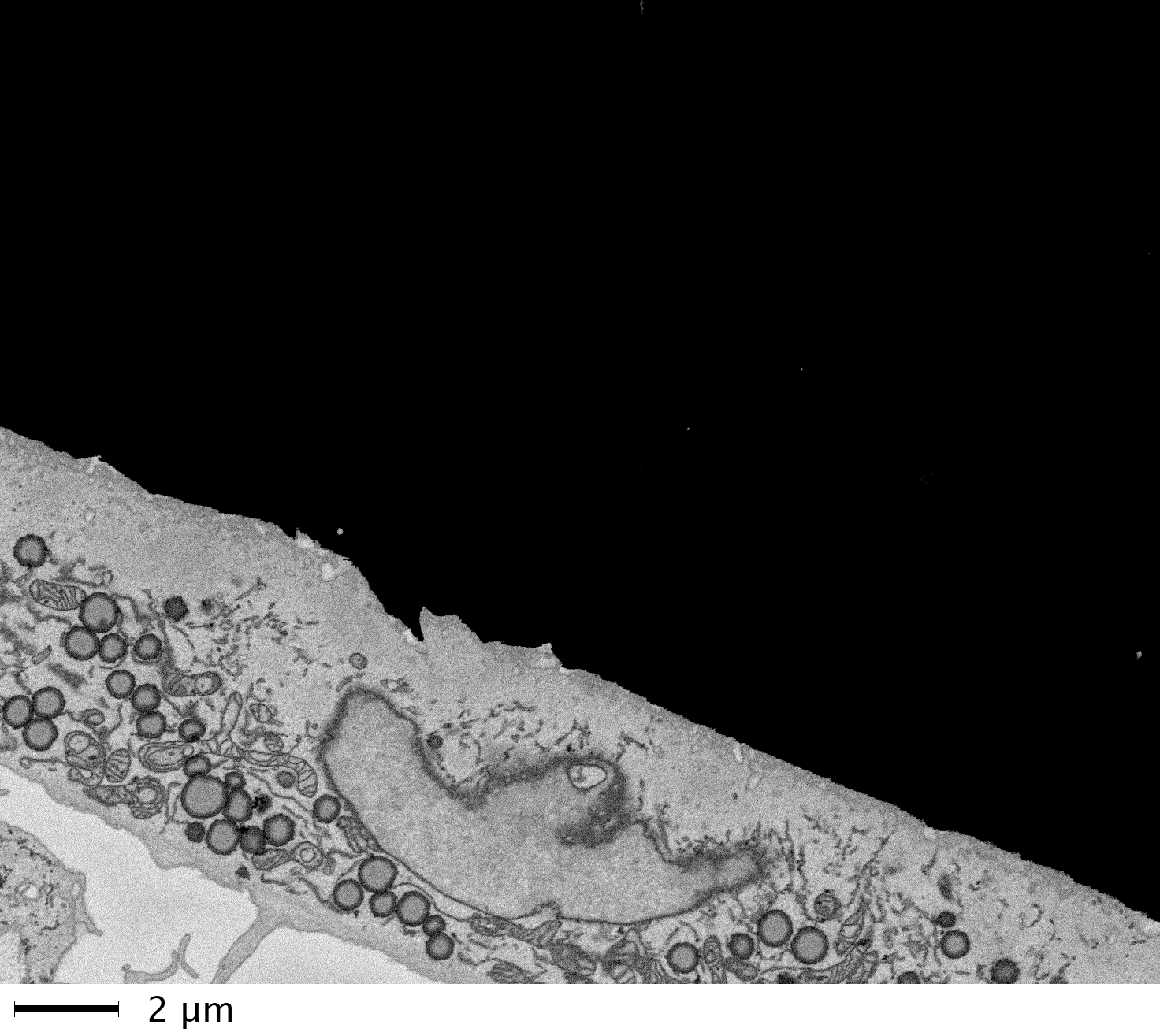

Supplement: Supplementary file 11 — Source Data for Expanded View and Appendix [file 44318_2025_423_MOESM11_ESM.zip › Figure_EV3/EV3A/Macrophage_2hChol+OA_0.33um.tif]

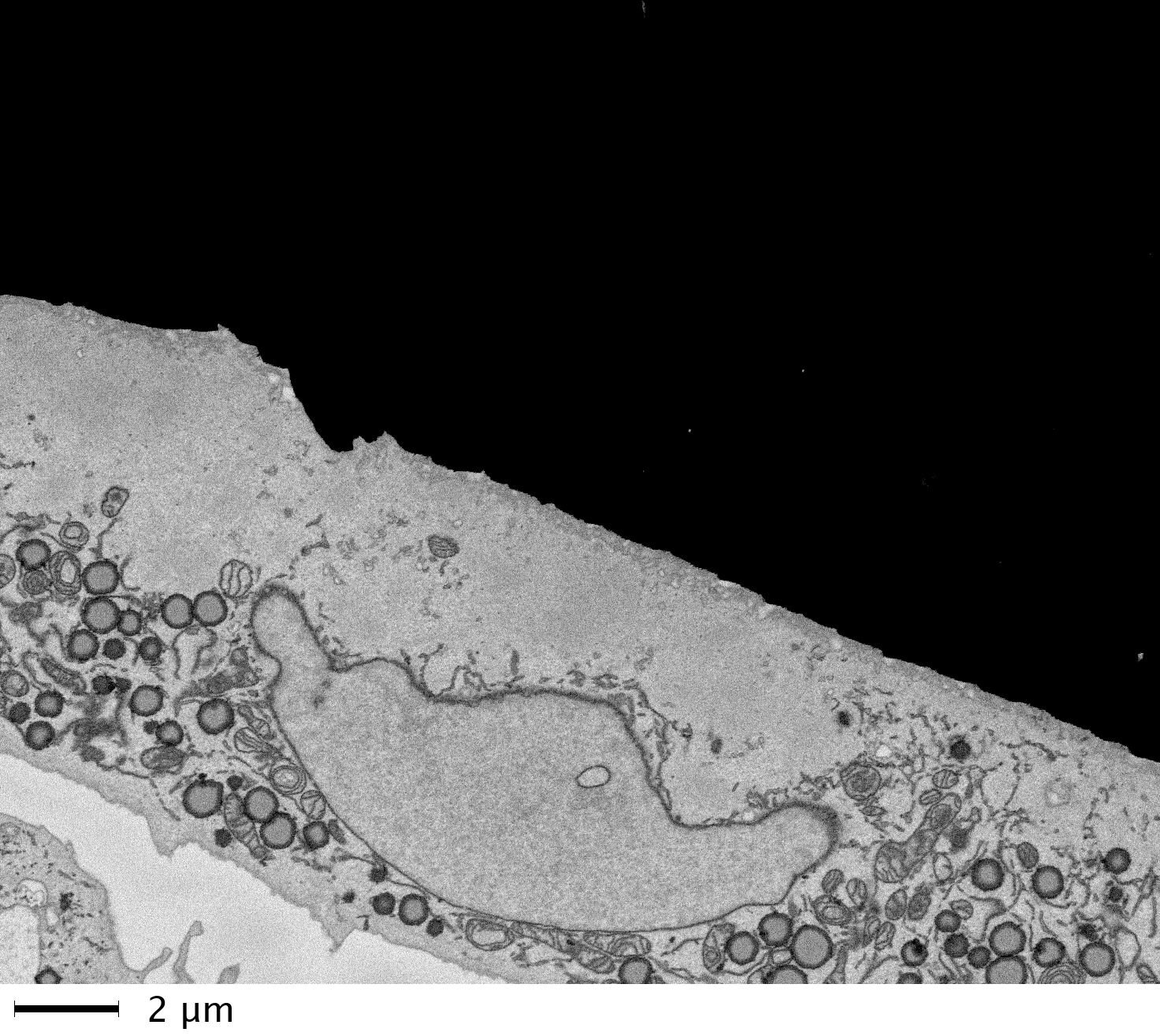

Supplement: Supplementary file 11 — Source Data for Expanded View and Appendix [file 44318_2025_423_MOESM11_ESM.zip › Figure_EV3/EV3A/Macrophage_2hChol+OA_0.63um.tif]

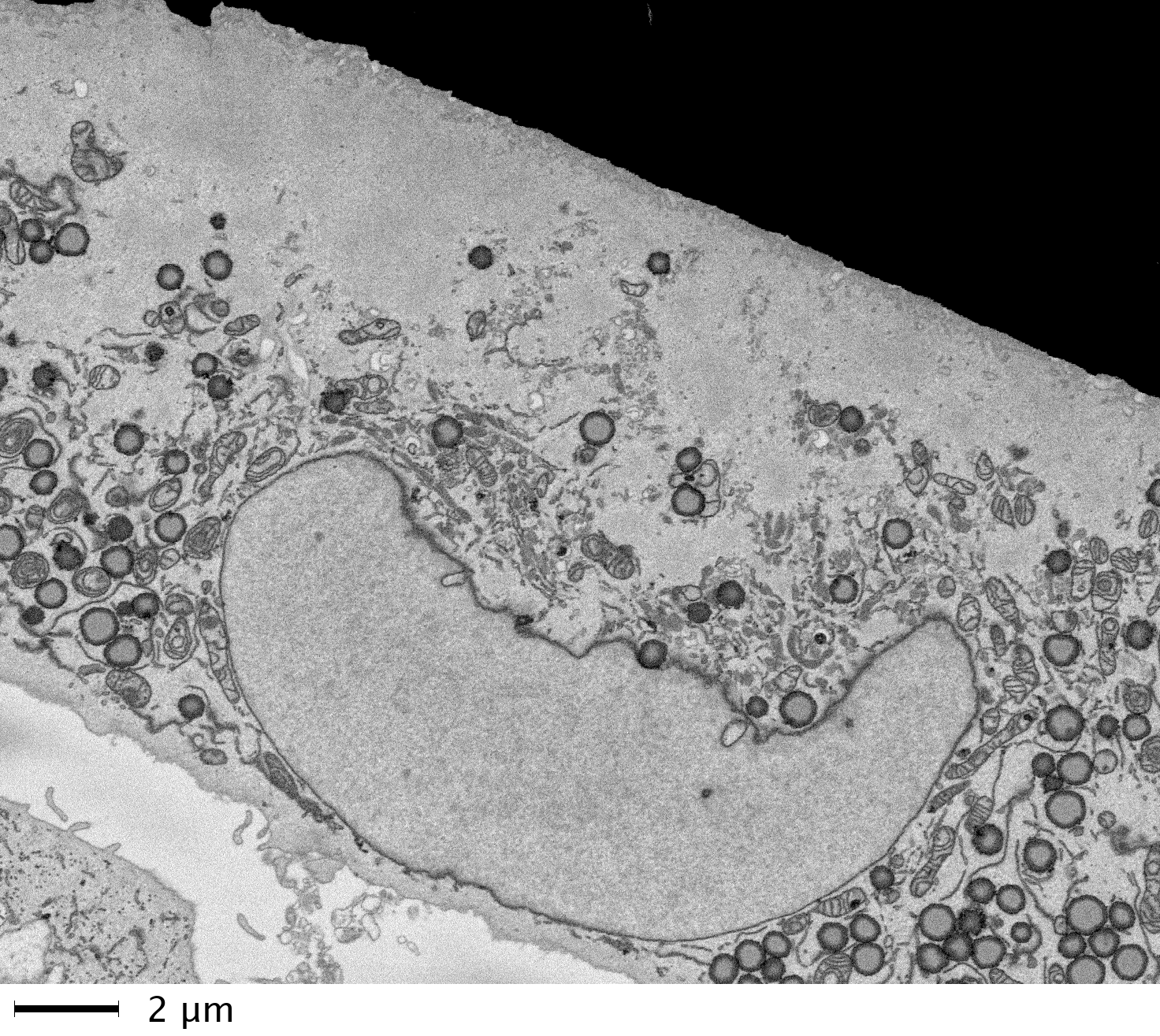

Supplement: Supplementary file 11 — Source Data for Expanded View and Appendix [file 44318_2025_423_MOESM11_ESM.zip › Figure_EV3/EV3A/Macrophage_2hChol+OA_1.35um.tif]

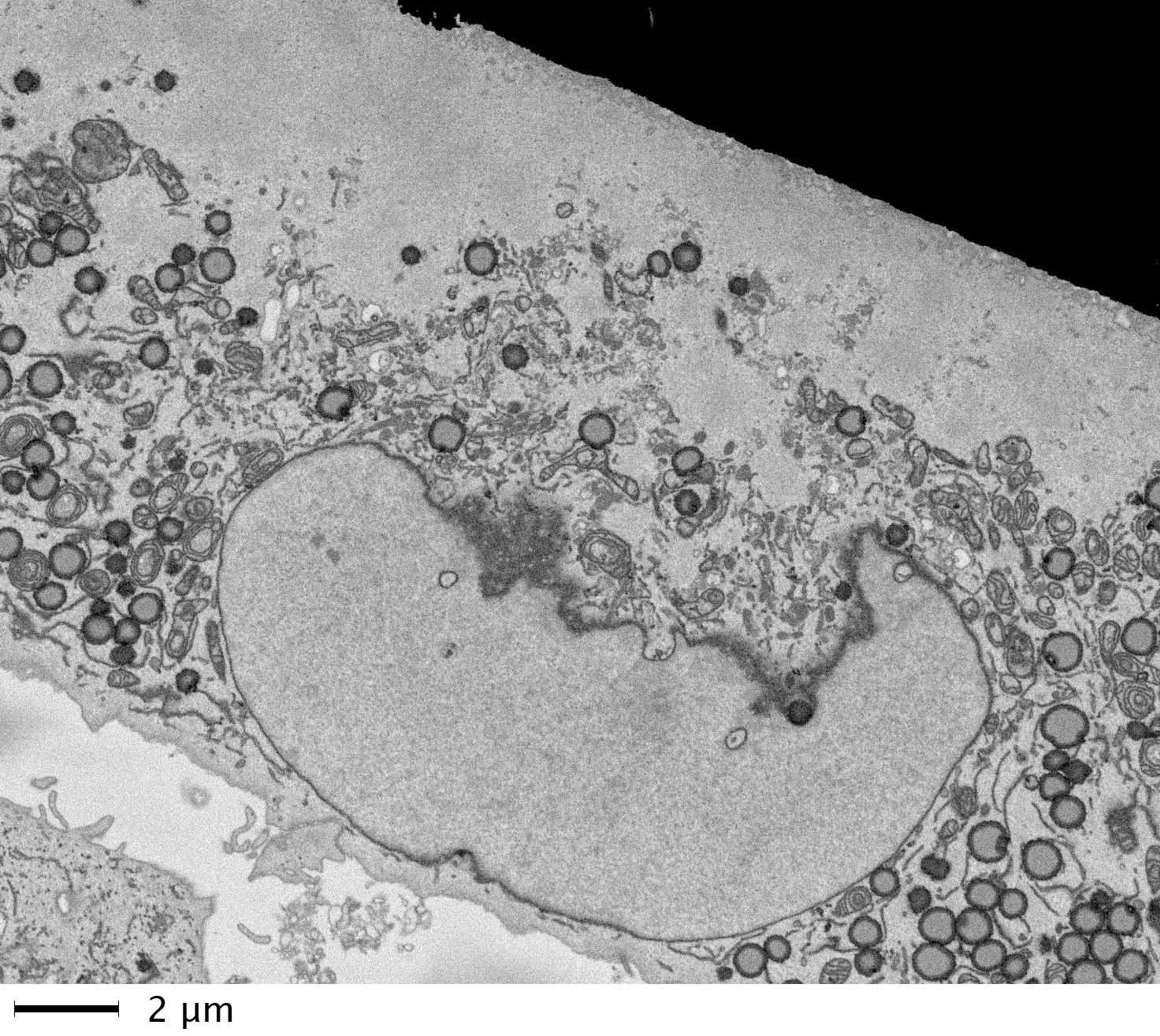

Supplement: Supplementary file 11 — Source Data for Expanded View and Appendix [file 44318_2025_423_MOESM11_ESM.zip › Figure_EV3/EV3A/Macrophage_2hChol+OA_1.50um.tif]

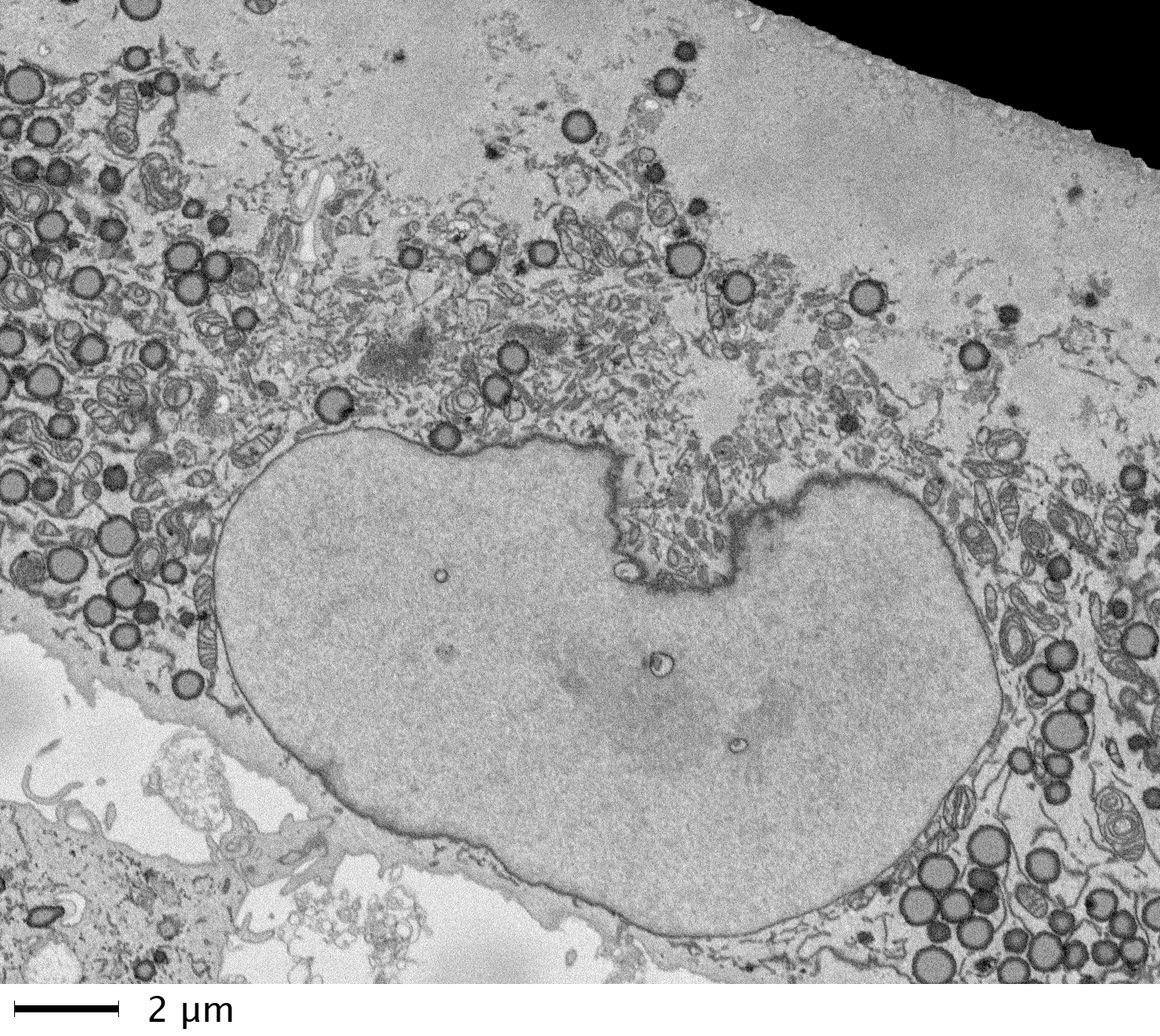

Supplement: Supplementary file 11 — Source Data for Expanded View and Appendix [file 44318_2025_423_MOESM11_ESM.zip › Figure_EV3/EV3A/Macrophage_2hChol+OA_1.80um.tif]

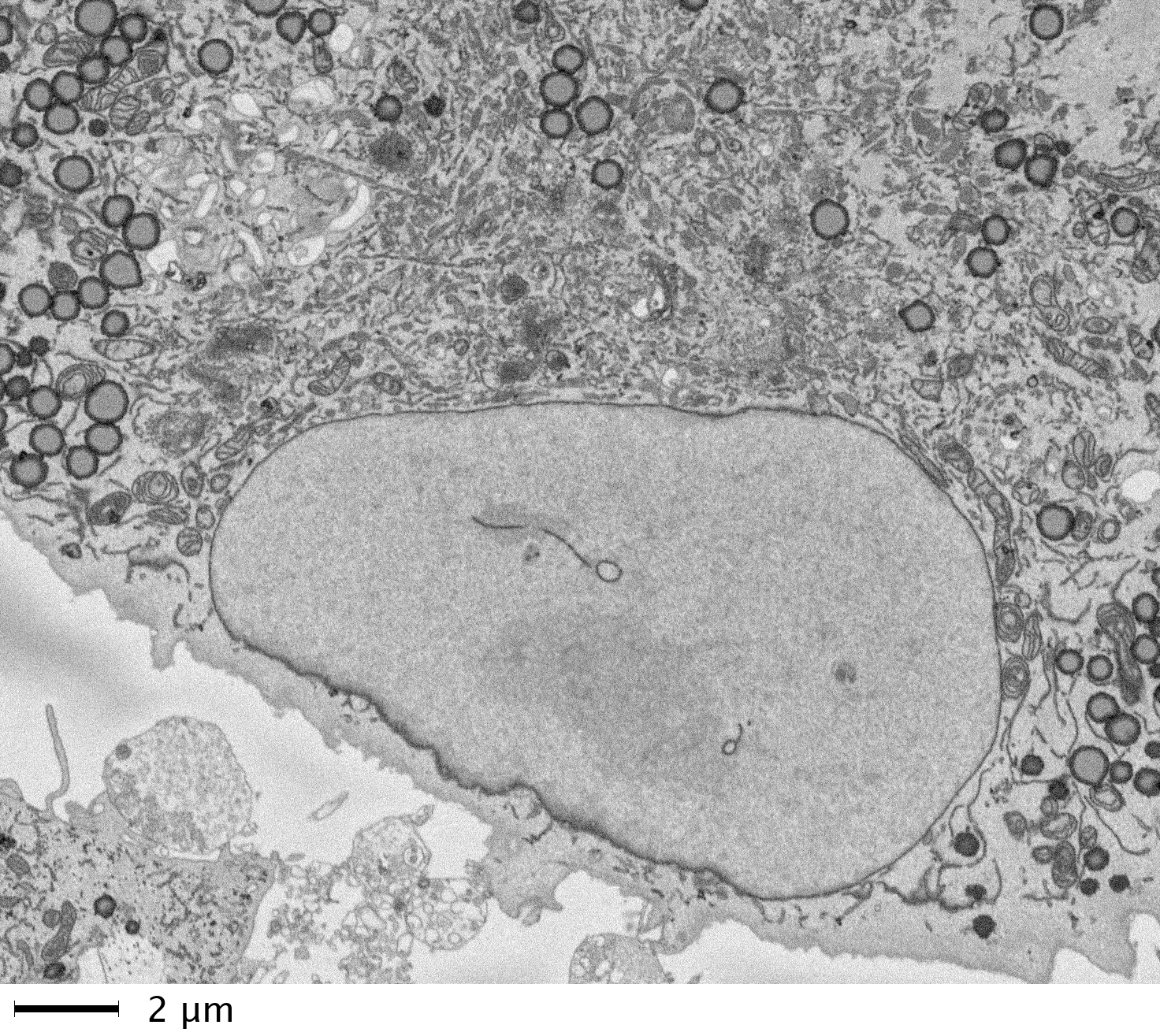

Supplement: Supplementary file 11 — Source Data for Expanded View and Appendix [file 44318_2025_423_MOESM11_ESM.zip › Figure_EV3/EV3A/Macrophage_2hChol+OA_2.55um.tif]

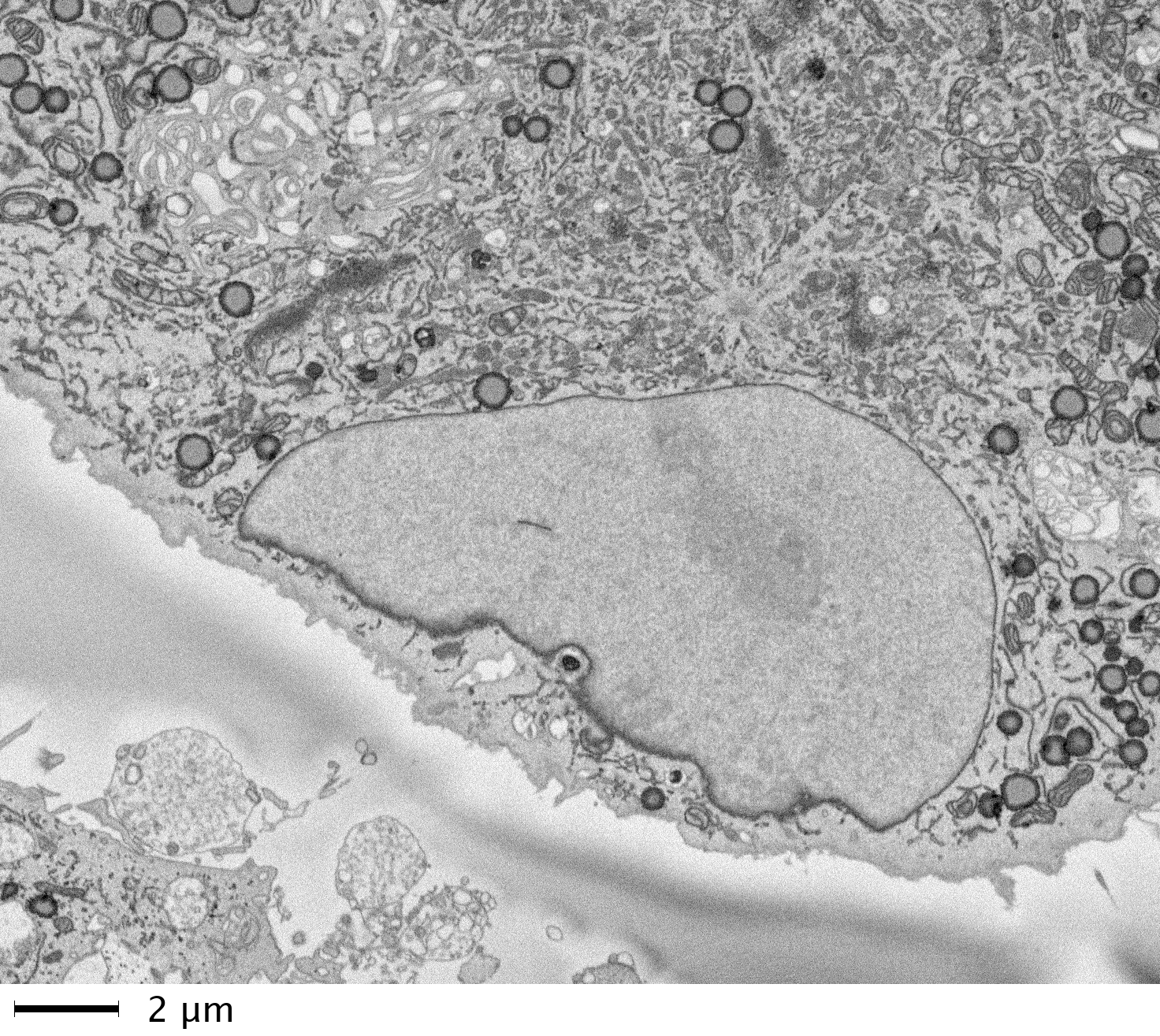

Supplement: Supplementary file 11 — Source Data for Expanded View and Appendix [file 44318_2025_423_MOESM11_ESM.zip › Figure_EV3/EV3A/Macrophage_2hChol+OA_3.30um.tif]

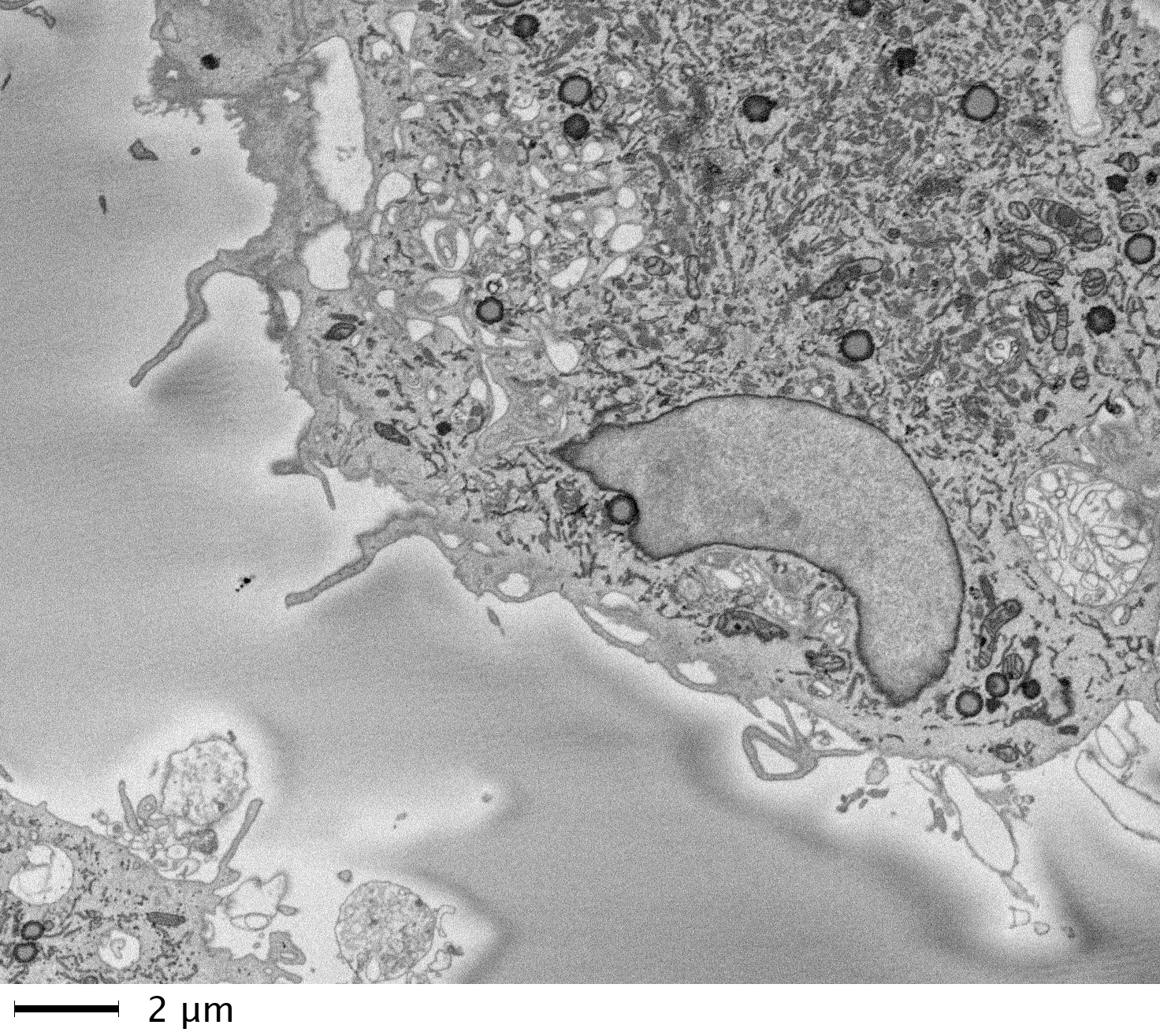

Supplement: Supplementary file 11 — Source Data for Expanded View and Appendix [file 44318_2025_423_MOESM11_ESM.zip › Figure_EV3/EV3A/Macrophage_2hChol+OA_4.35um.tif]

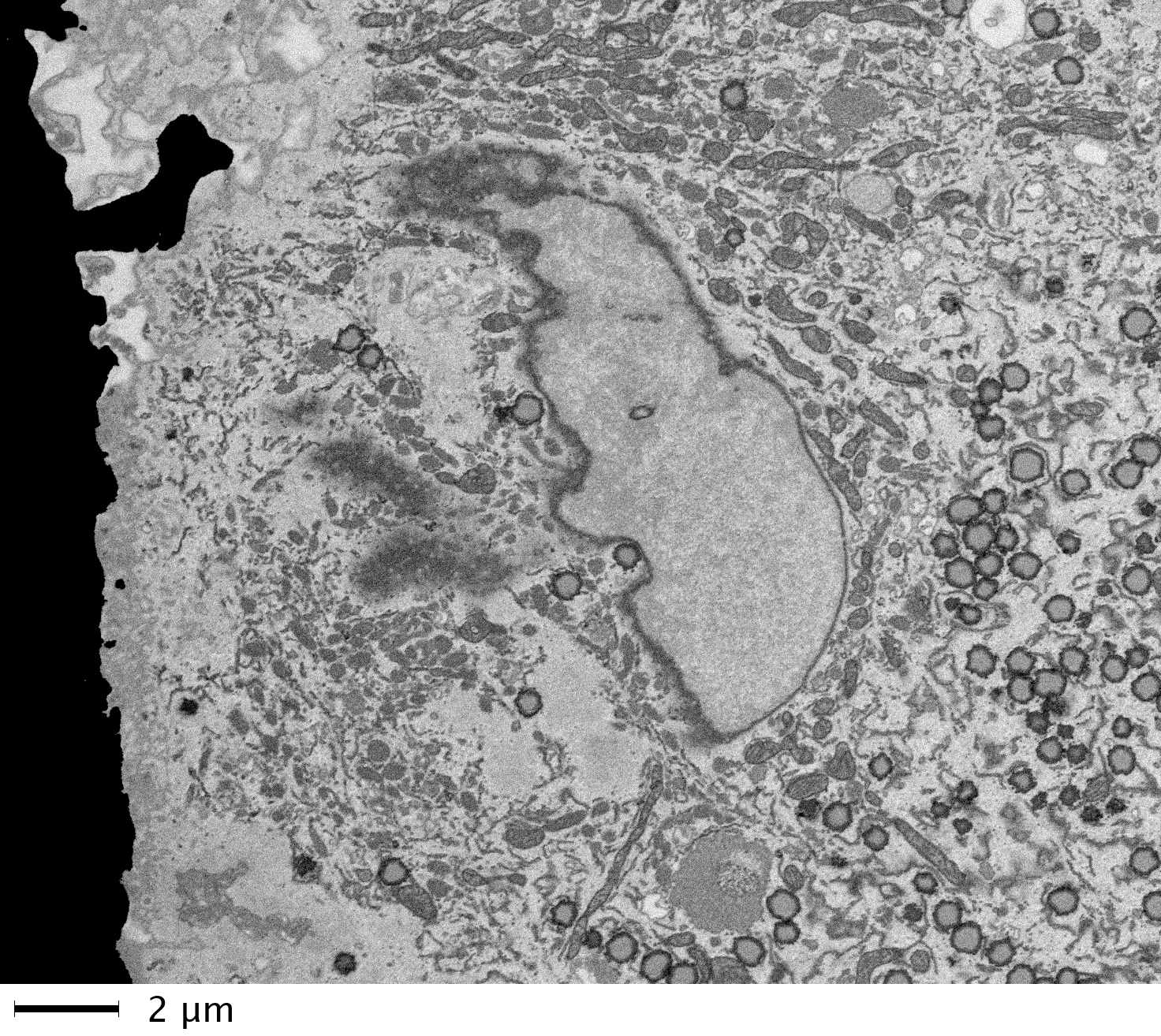

Supplement: Supplementary file 11 — Source Data for Expanded View and Appendix [file 44318_2025_423_MOESM11_ESM.zip › Figure_EV3/EV3B/Macrophage_2hChol+OA_0.42um.tif]

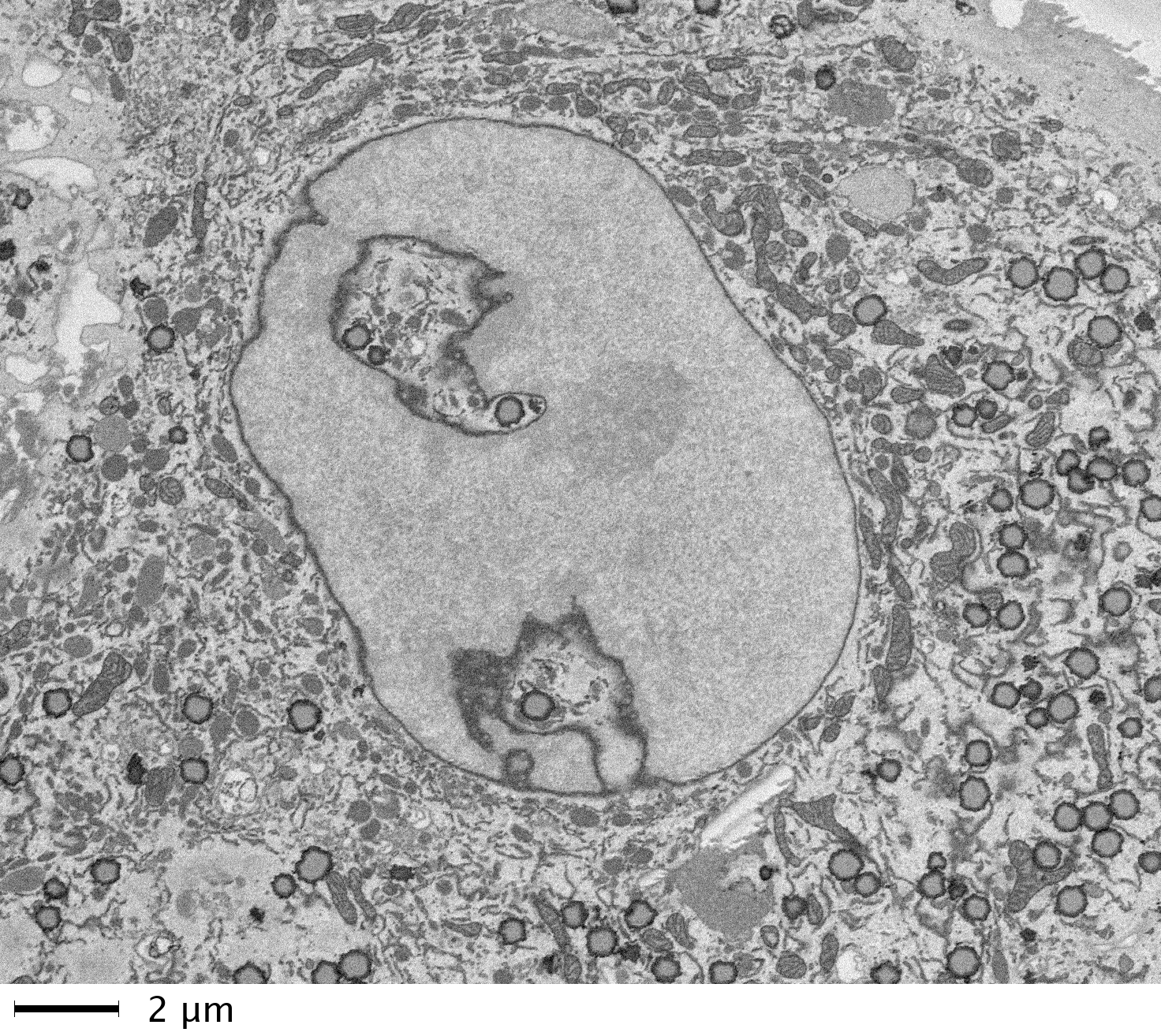

Supplement: Supplementary file 11 — Source Data for Expanded View and Appendix [file 44318_2025_423_MOESM11_ESM.zip › Figure_EV3/EV3B/Macrophage_2hChol+OA_0.66um.tif]

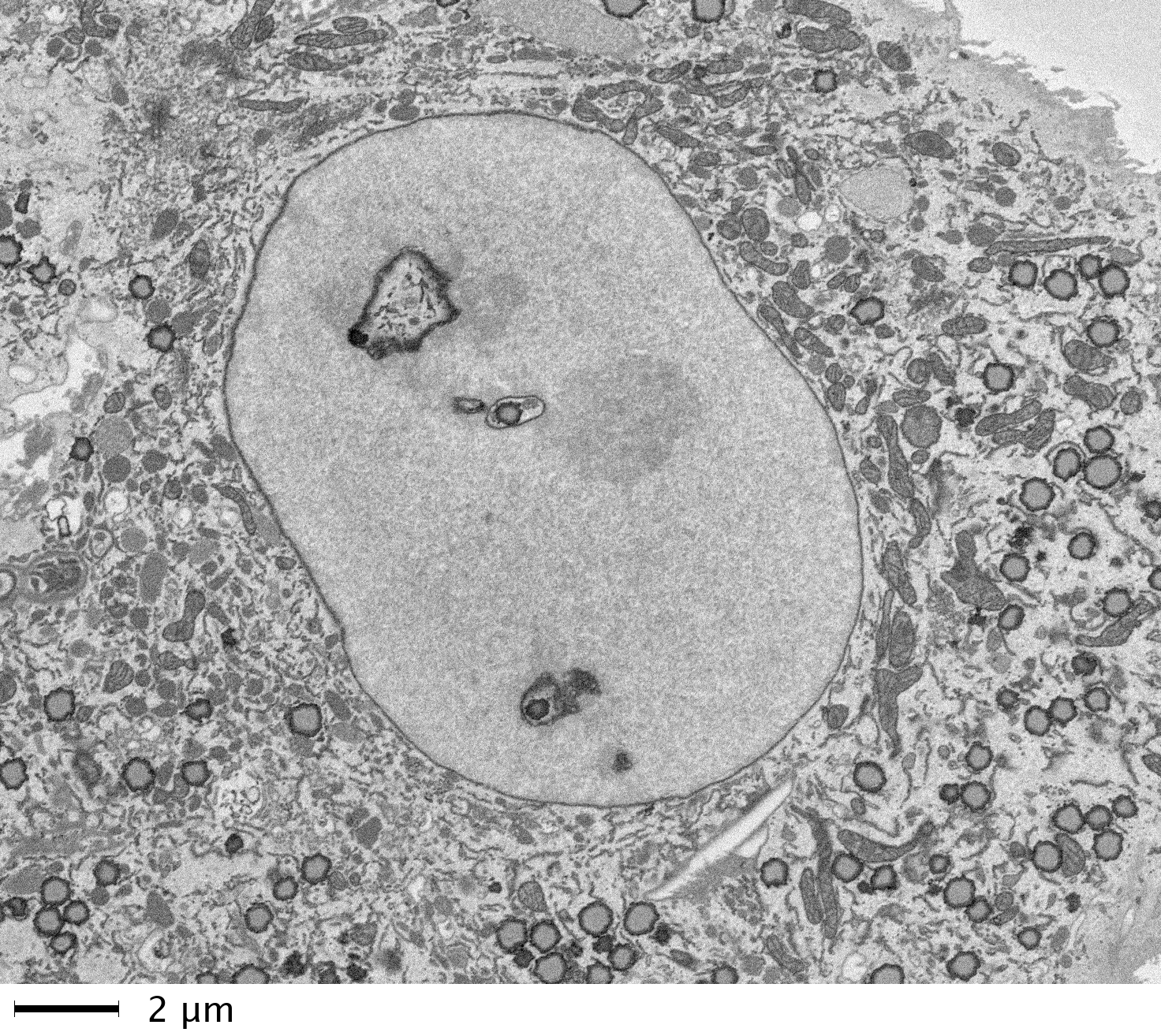

Supplement: Supplementary file 11 — Source Data for Expanded View and Appendix [file 44318_2025_423_MOESM11_ESM.zip › Figure_EV3/EV3B/Macrophage_2hChol+OA_0.78um.tif]

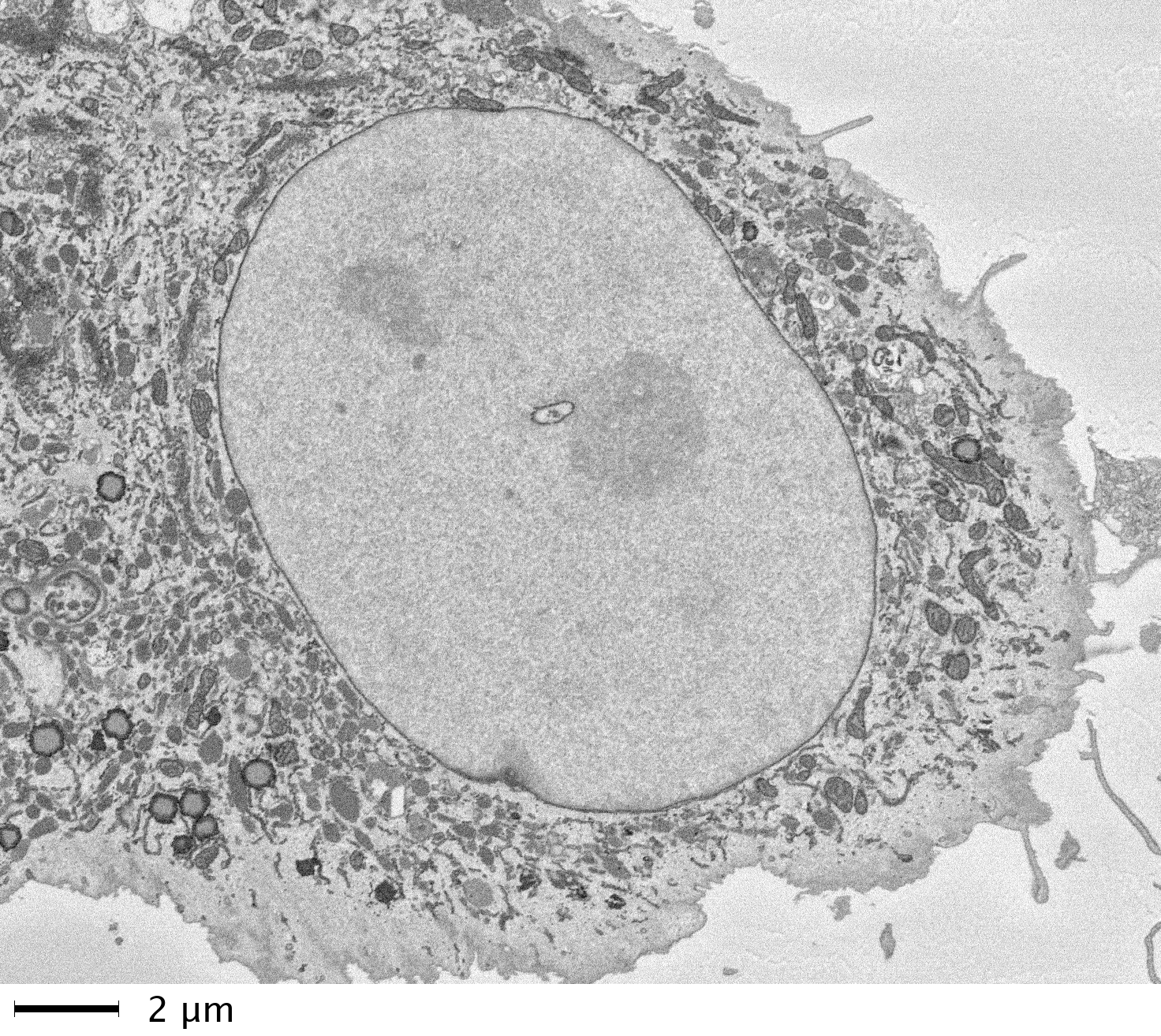

Supplement: Supplementary file 11 — Source Data for Expanded View and Appendix [file 44318_2025_423_MOESM11_ESM.zip › Figure_EV3/EV3B/Macrophage_2hChol+OA_1.29um.tif]

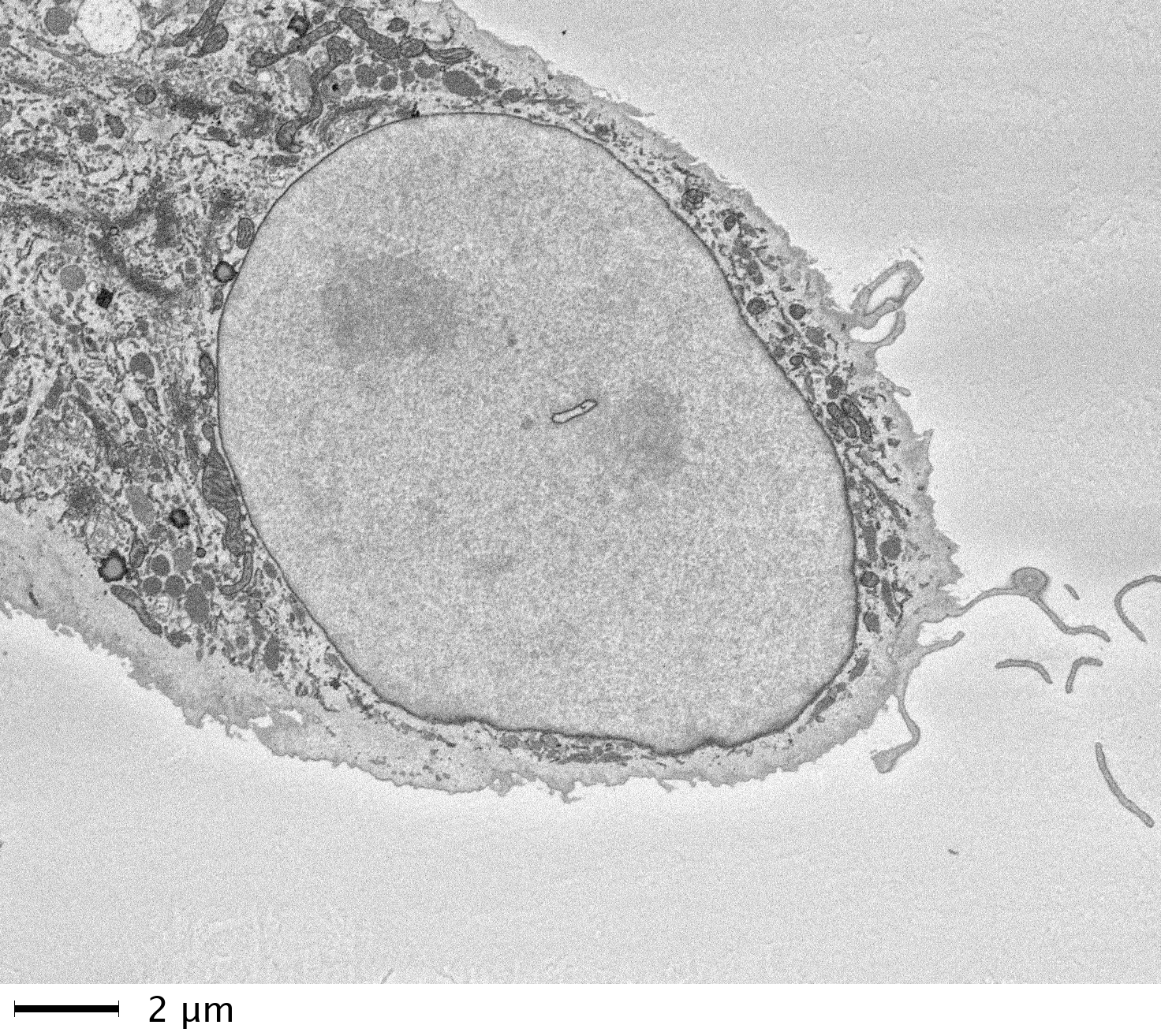

Supplement: Supplementary file 11 — Source Data for Expanded View and Appendix [file 44318_2025_423_MOESM11_ESM.zip › Figure_EV3/EV3B/Macrophage_2hChol+OA_1.74um.tif]

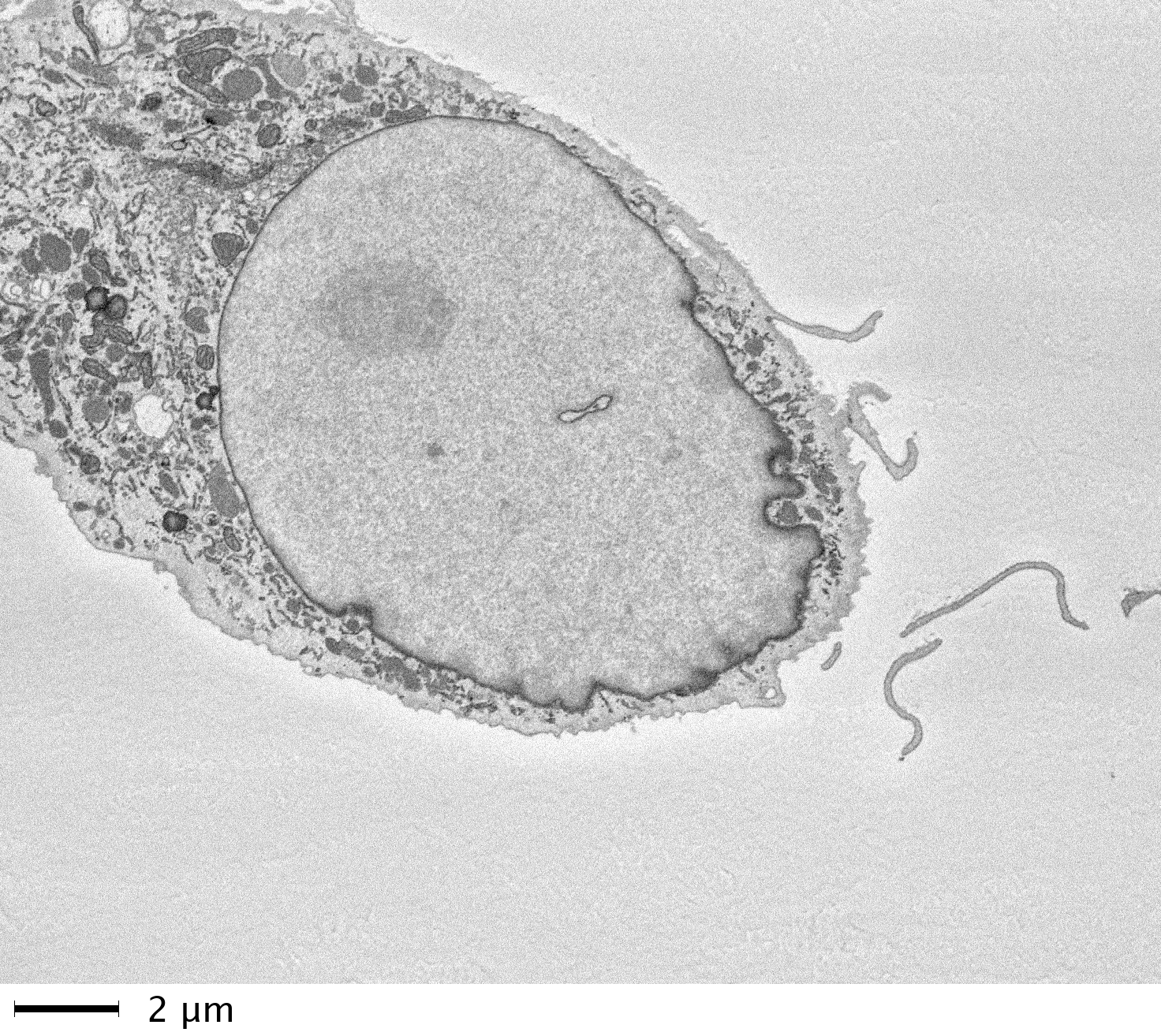

Supplement: Supplementary file 11 — Source Data for Expanded View and Appendix [file 44318_2025_423_MOESM11_ESM.zip › Figure_EV3/EV3B/Macrophage_2hChol+OA_2.04um.tif]

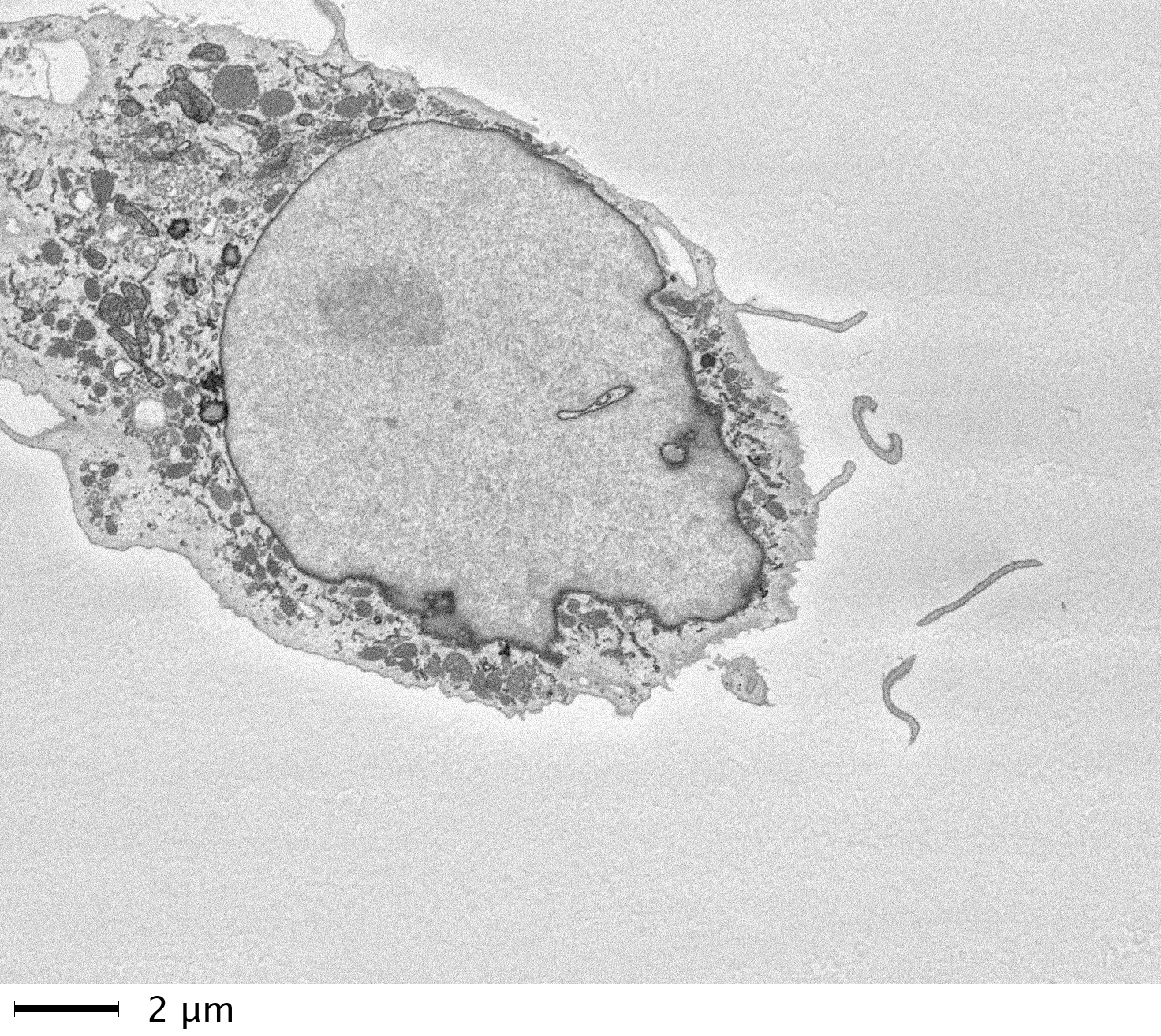

Supplement: Supplementary file 11 — Source Data for Expanded View and Appendix [file 44318_2025_423_MOESM11_ESM.zip › Figure_EV3/EV3B/Macrophage_2hChol+OA_2.22um.tif]

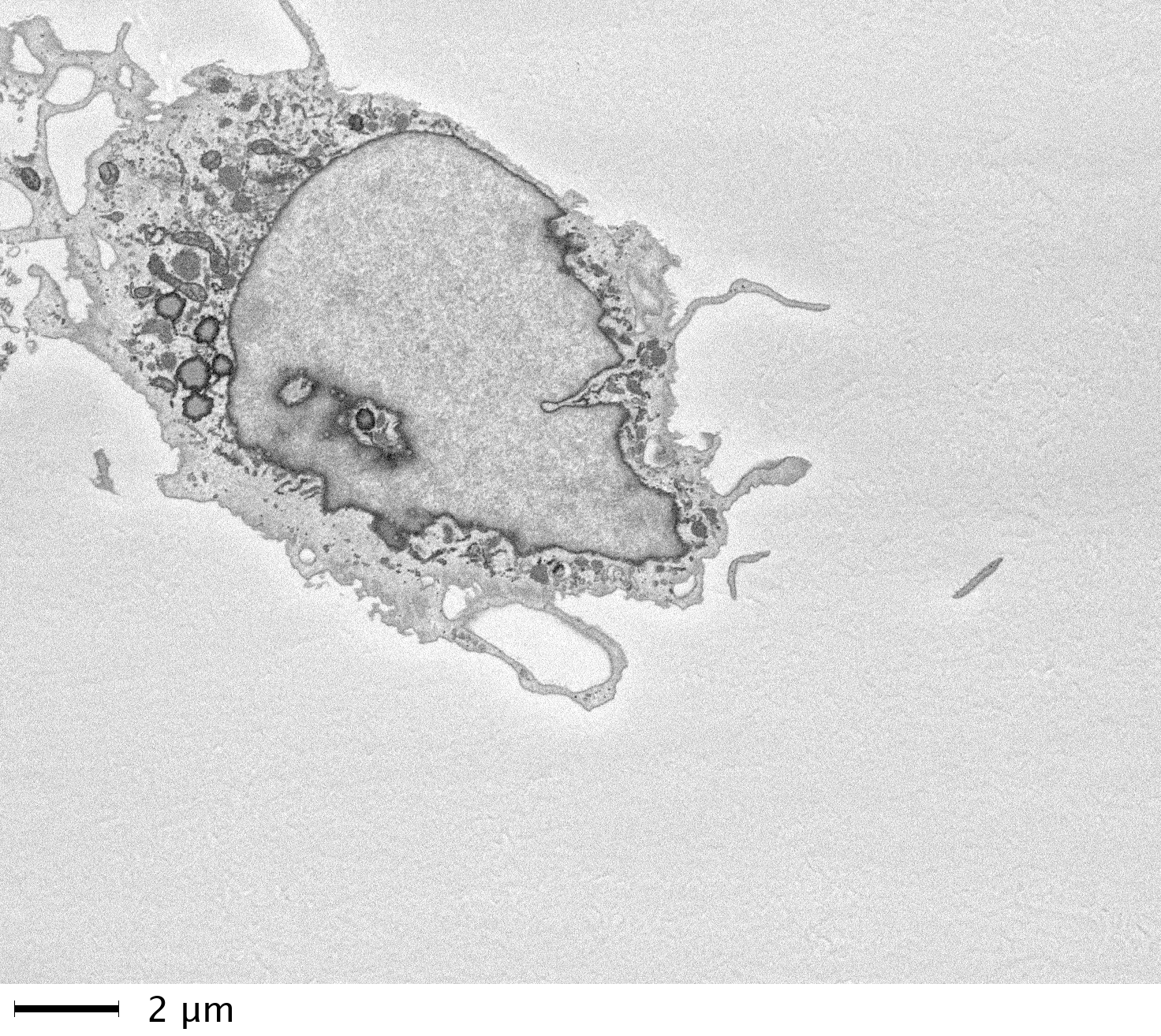

Supplement: Supplementary file 11 — Source Data for Expanded View and Appendix [file 44318_2025_423_MOESM11_ESM.zip › Figure_EV3/EV3B/Macrophage_2hChol+OA_2.52um.tif]

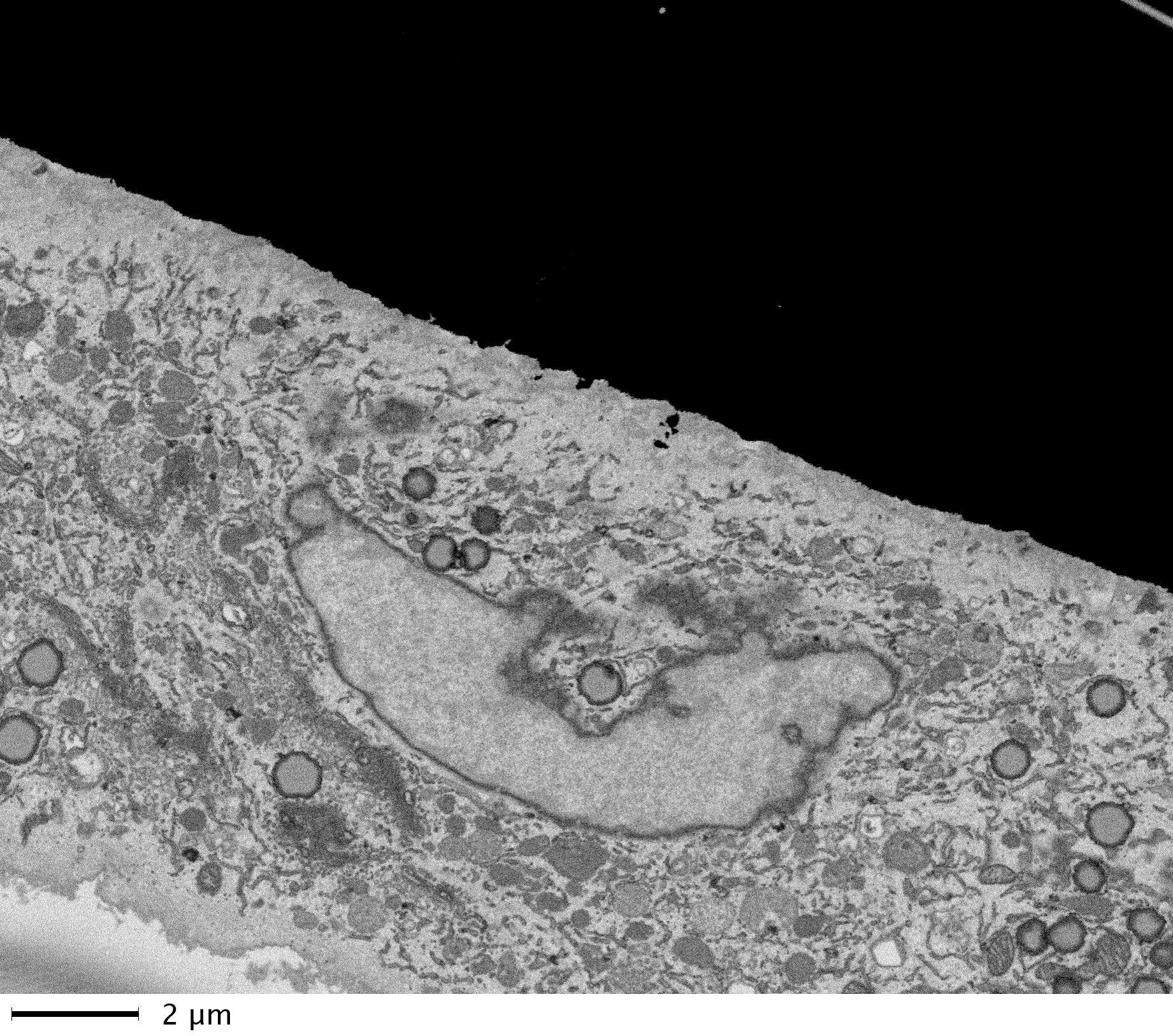

Supplement: Supplementary file 11 — Source Data for Expanded View and Appendix [file 44318_2025_423_MOESM11_ESM.zip › Figure_EV3/EV3C/Macrophage_2hChol+OA_0.39um.tif]

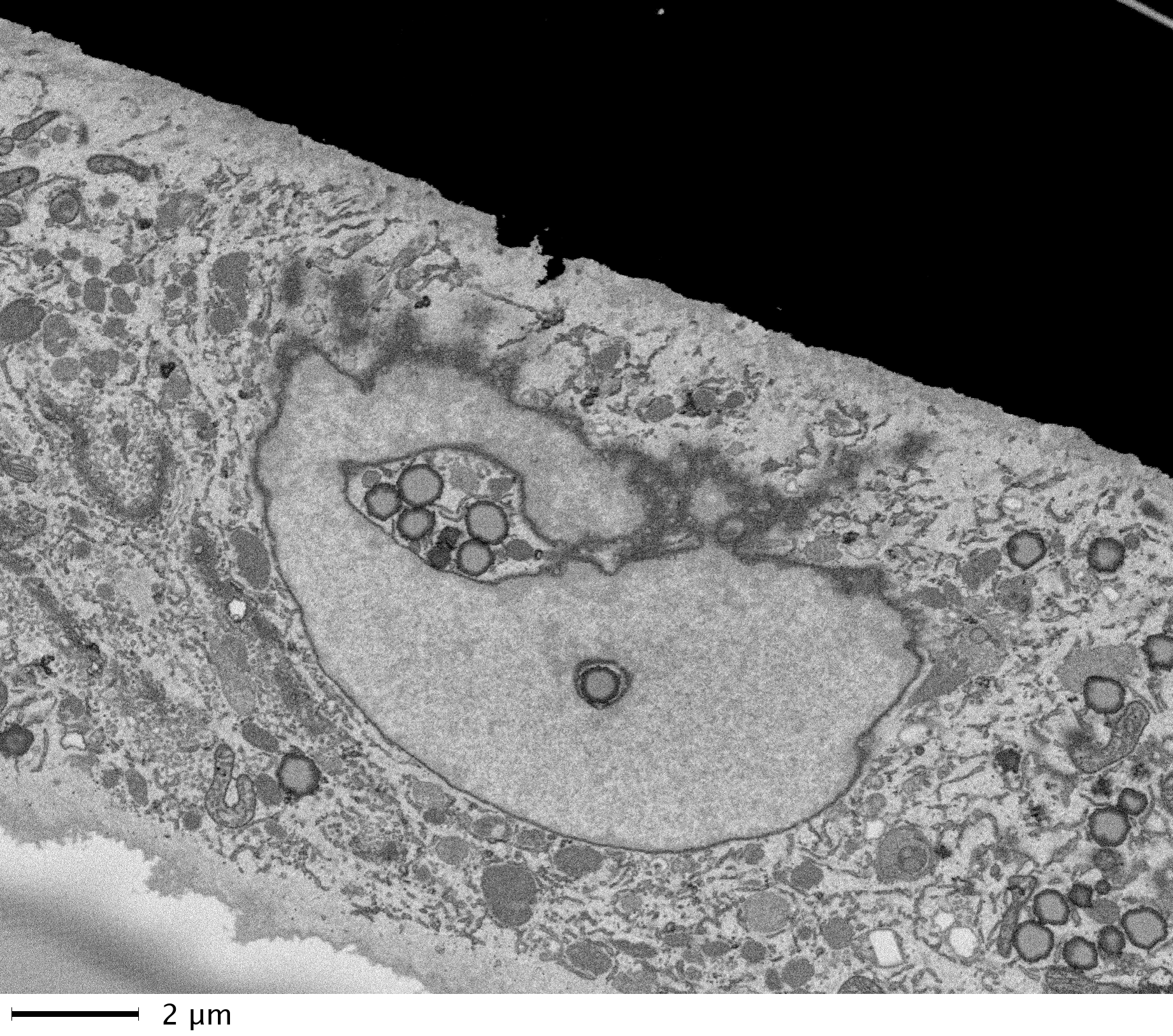

Supplement: Supplementary file 11 — Source Data for Expanded View and Appendix [file 44318_2025_423_MOESM11_ESM.zip › Figure_EV3/EV3C/Macrophage_2hChol+OA_0.60um.tif]

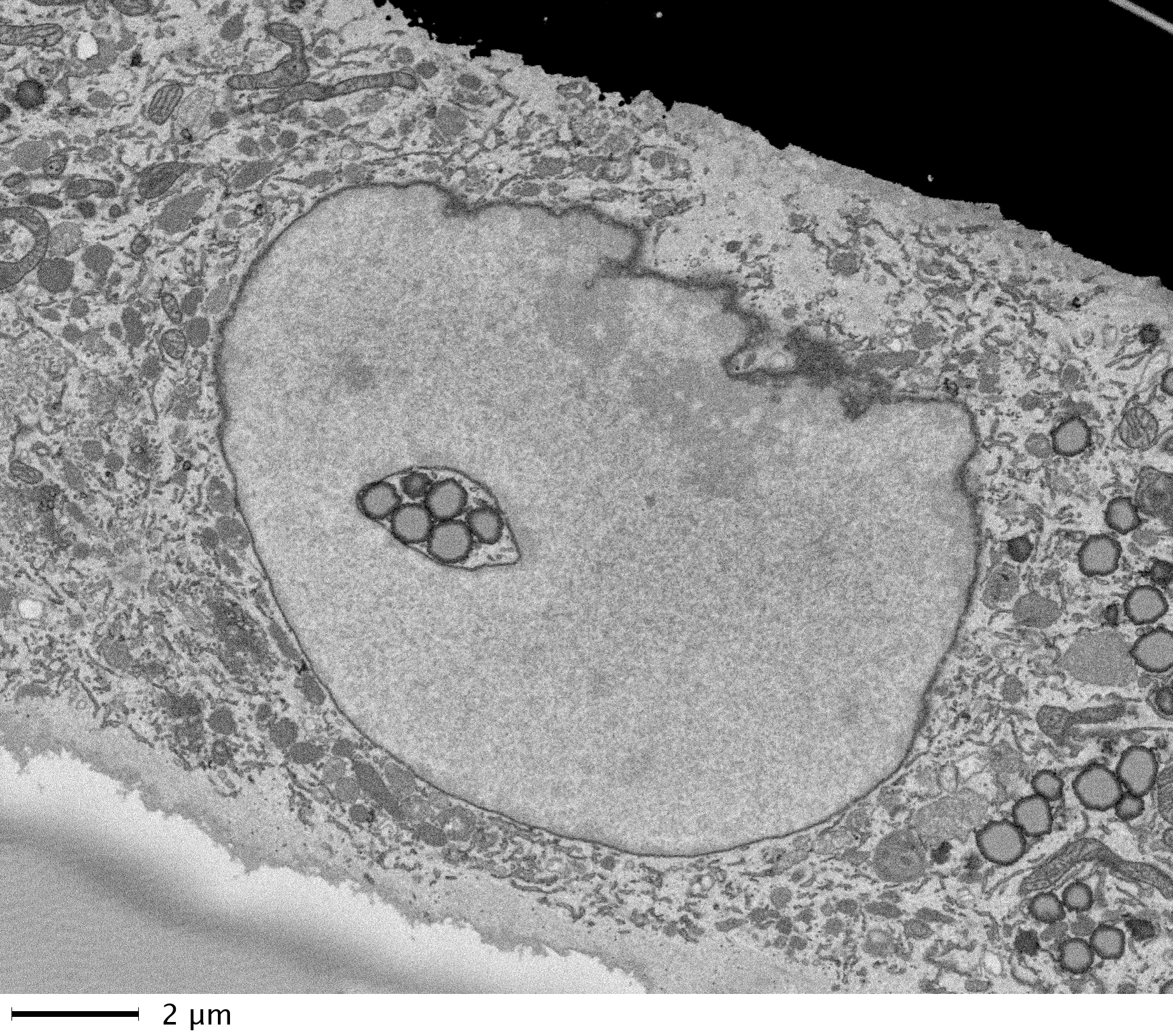

Supplement: Supplementary file 11 — Source Data for Expanded View and Appendix [file 44318_2025_423_MOESM11_ESM.zip › Figure_EV3/EV3C/Macrophage_2hChol+OA_0.90um.tif]

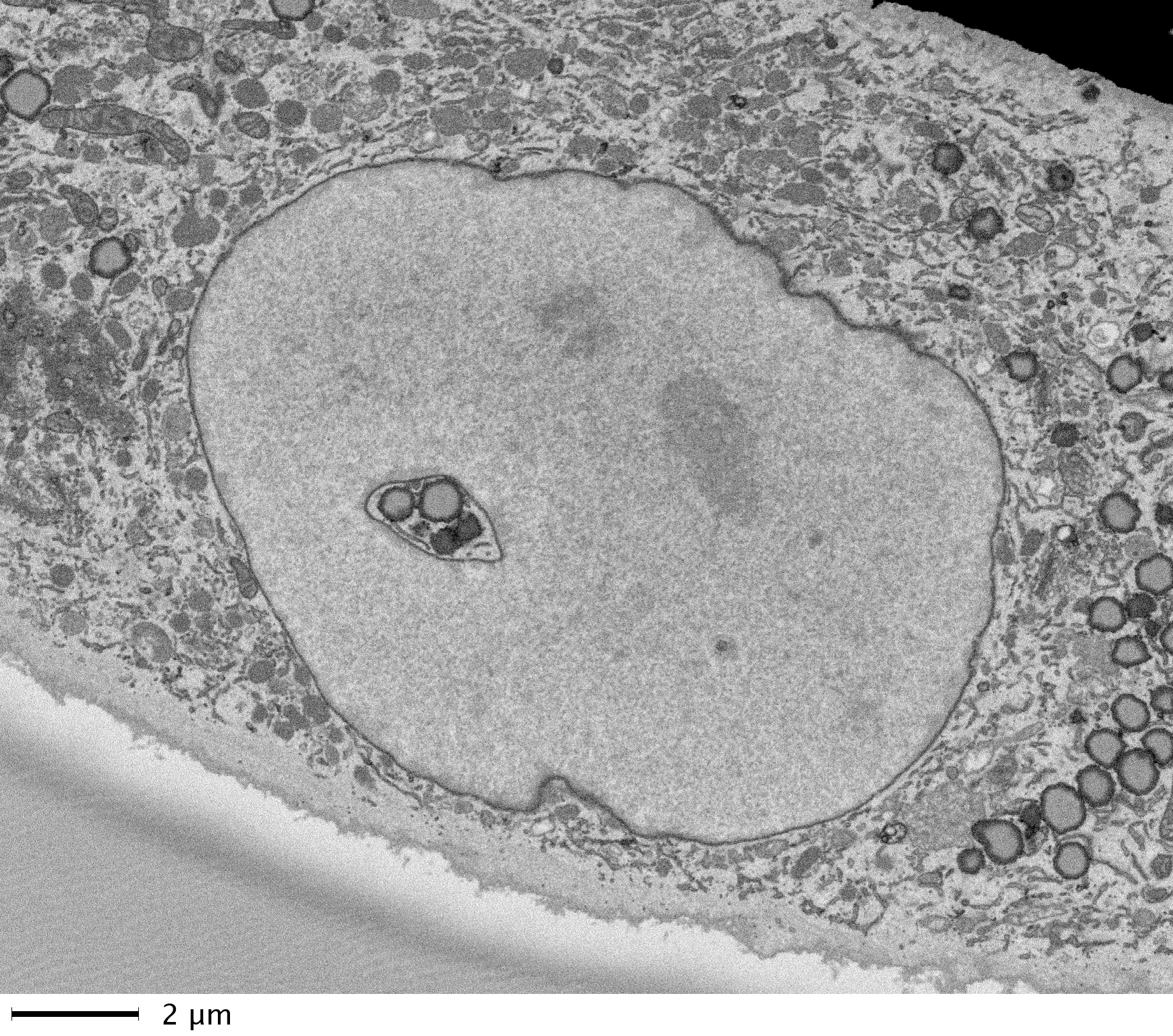

Supplement: Supplementary file 11 — Source Data for Expanded View and Appendix [file 44318_2025_423_MOESM11_ESM.zip › Figure_EV3/EV3C/Macrophage_2hChol+OA_1.20um.tif]

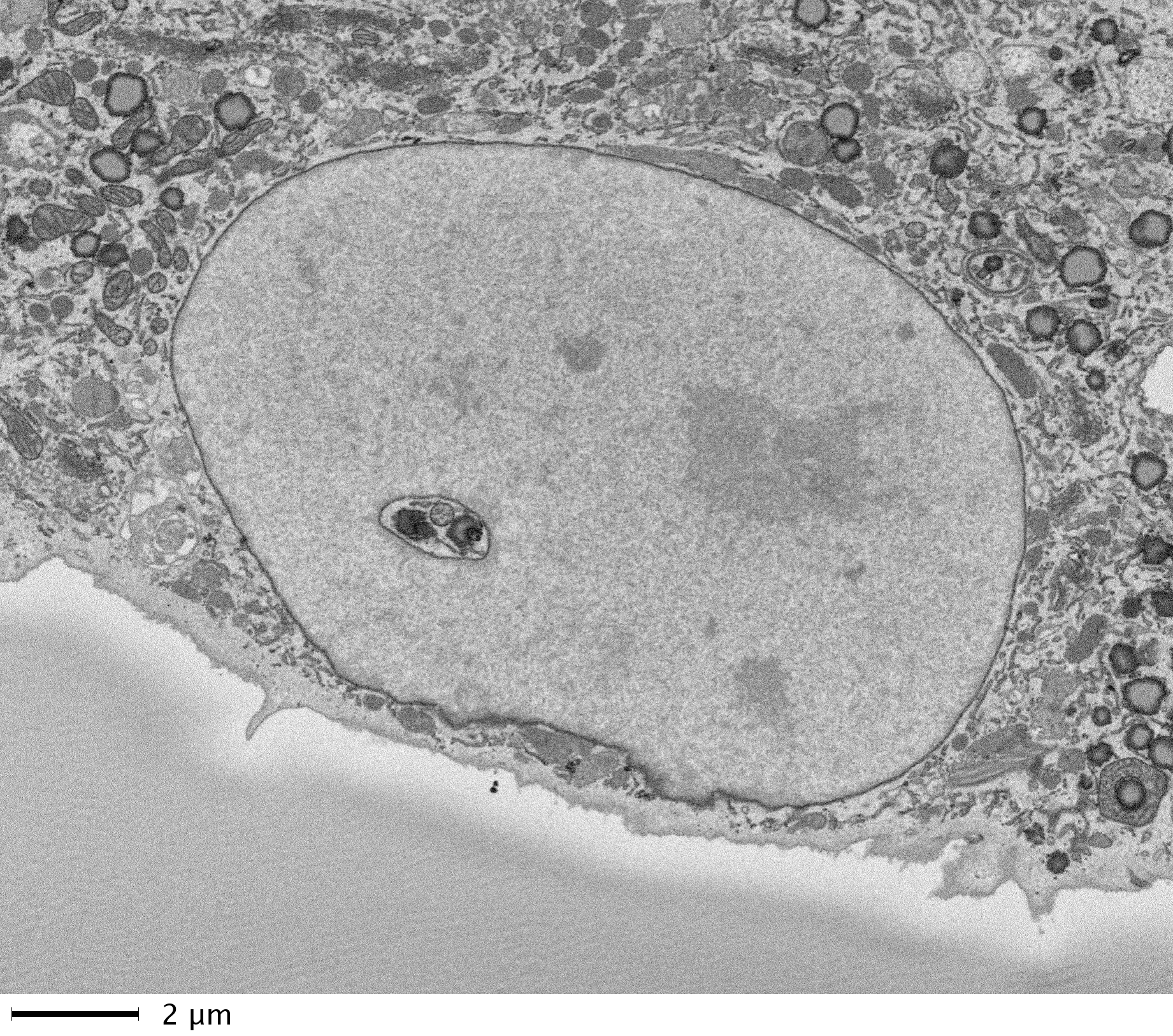

Supplement: Supplementary file 11 — Source Data for Expanded View and Appendix [file 44318_2025_423_MOESM11_ESM.zip › Figure_EV3/EV3C/Macrophage_2hChol+OA_1.80um.tif]

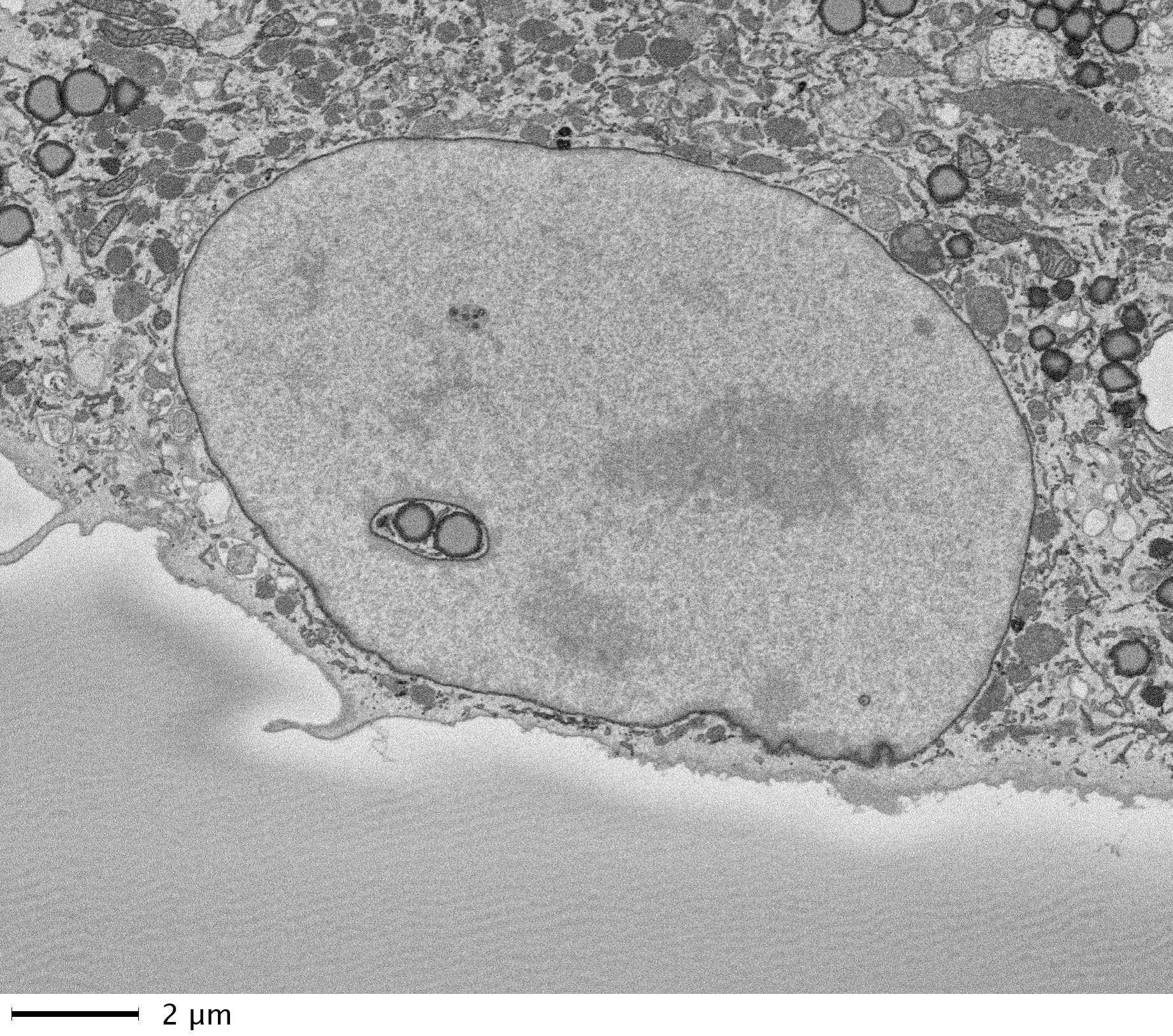

Supplement: Supplementary file 11 — Source Data for Expanded View and Appendix [file 44318_2025_423_MOESM11_ESM.zip › Figure_EV3/EV3C/Macrophage_2hChol+OA_2.28um.tif]

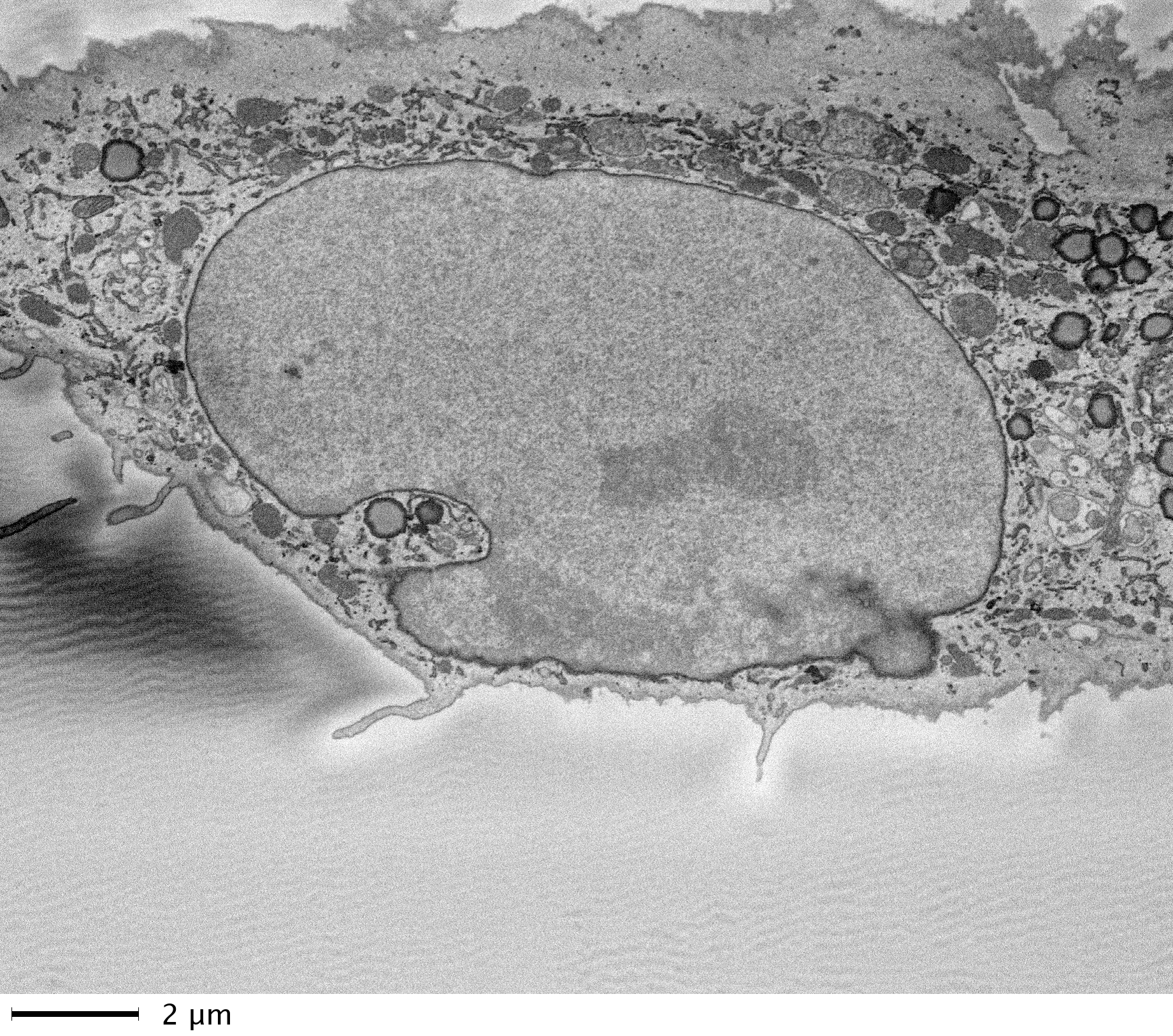

Supplement: Supplementary file 11 — Source Data for Expanded View and Appendix [file 44318_2025_423_MOESM11_ESM.zip › Figure_EV3/EV3C/Macrophage_2hChol+OA_2.76um.tif]

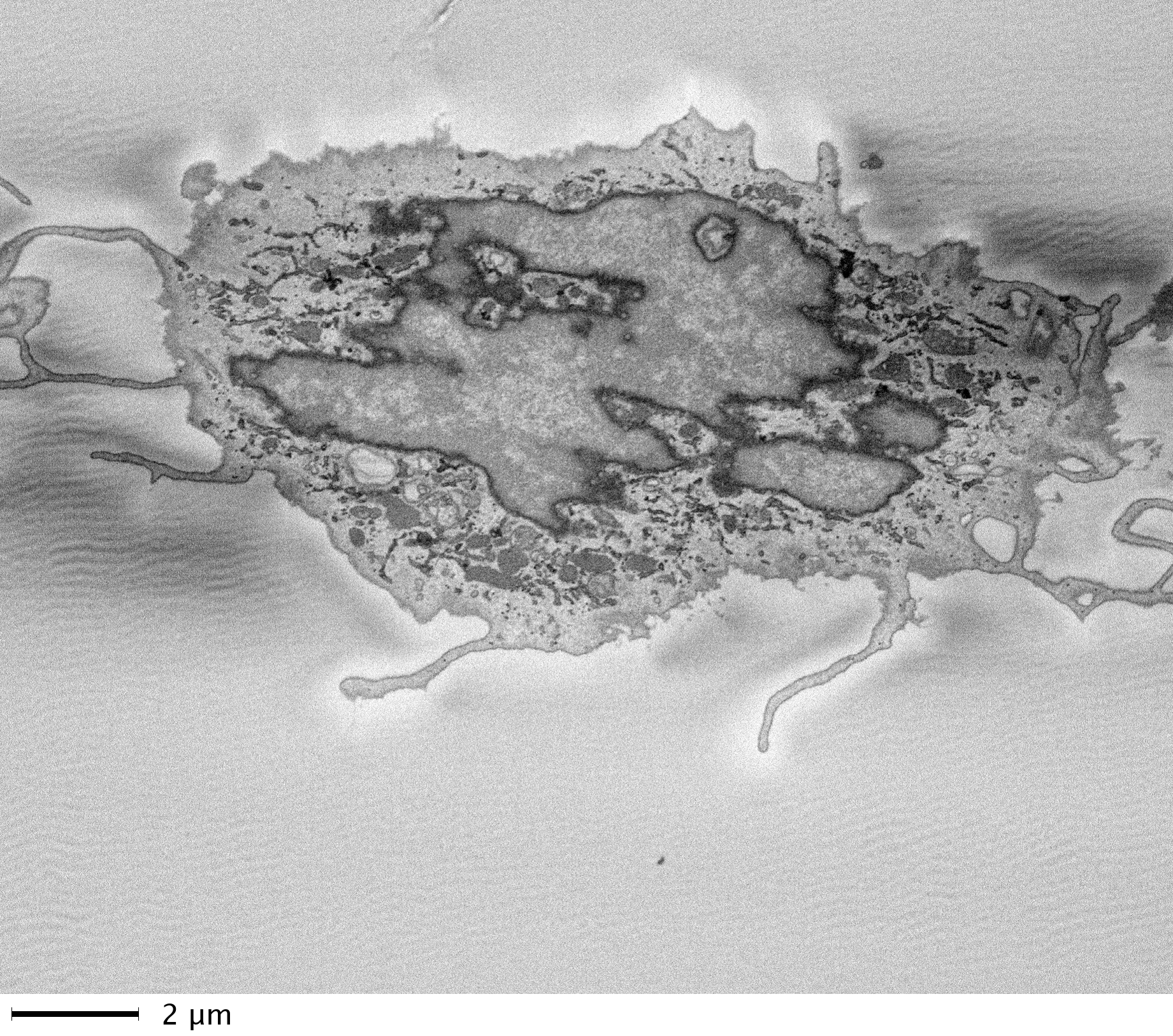

Supplement: Supplementary file 11 — Source Data for Expanded View and Appendix [file 44318_2025_423_MOESM11_ESM.zip › Figure_EV3/EV3C/Macrophage_2hChol+OA_3.52um.tif]

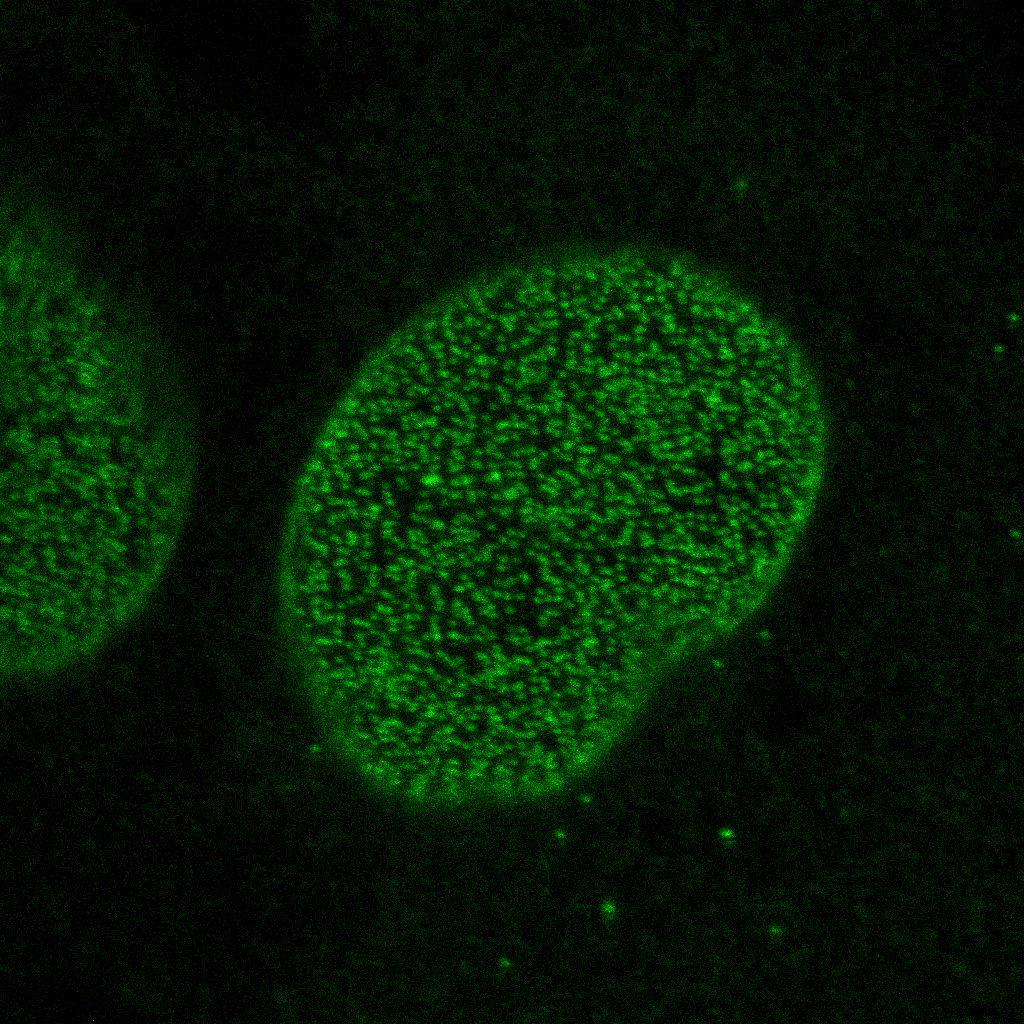

Supplement: Supplementary file 11 — Source Data for Expanded View and Appendix [file 44318_2025_423_MOESM11_ESM.zip › Figure_EV5/EV5E/A431-NUP93_0minTNFa.png]

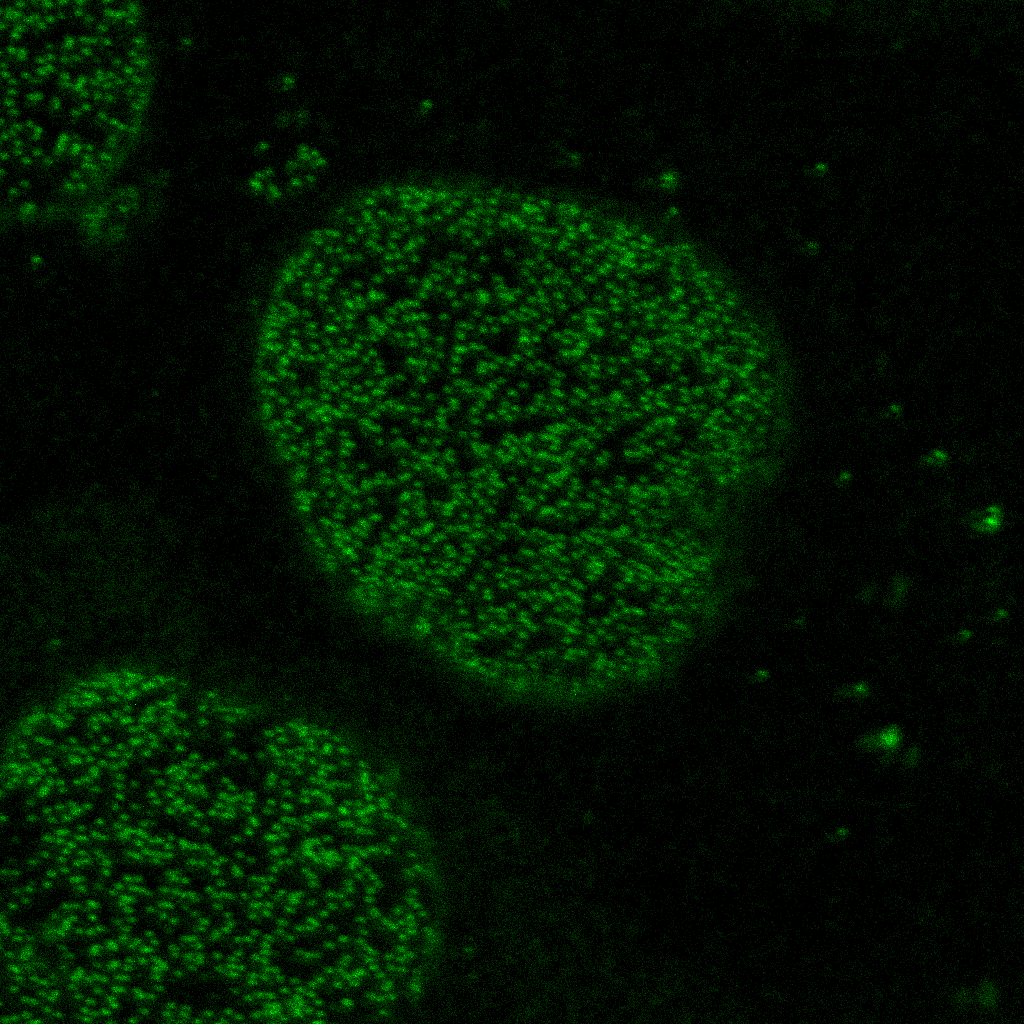

Supplement: Supplementary file 11 — Source Data for Expanded View and Appendix [file 44318_2025_423_MOESM11_ESM.zip › Figure_EV5/EV5E/A431-NUP93_30minTNFa.png]

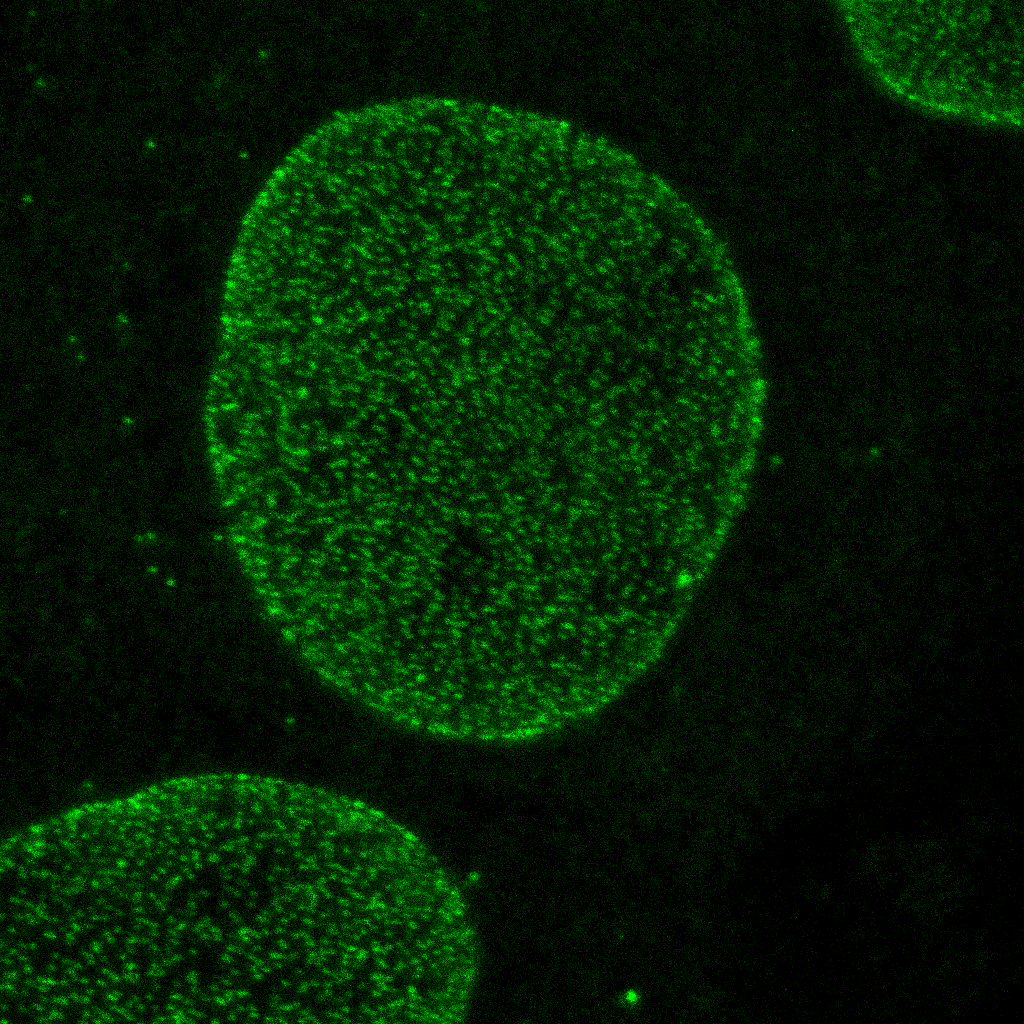

Supplement: Supplementary file 11 — Source Data for Expanded View and Appendix [file 44318_2025_423_MOESM11_ESM.zip › Figure_EV5/EV5E/A431-NUP93_30minTNFa+SOATi.png]

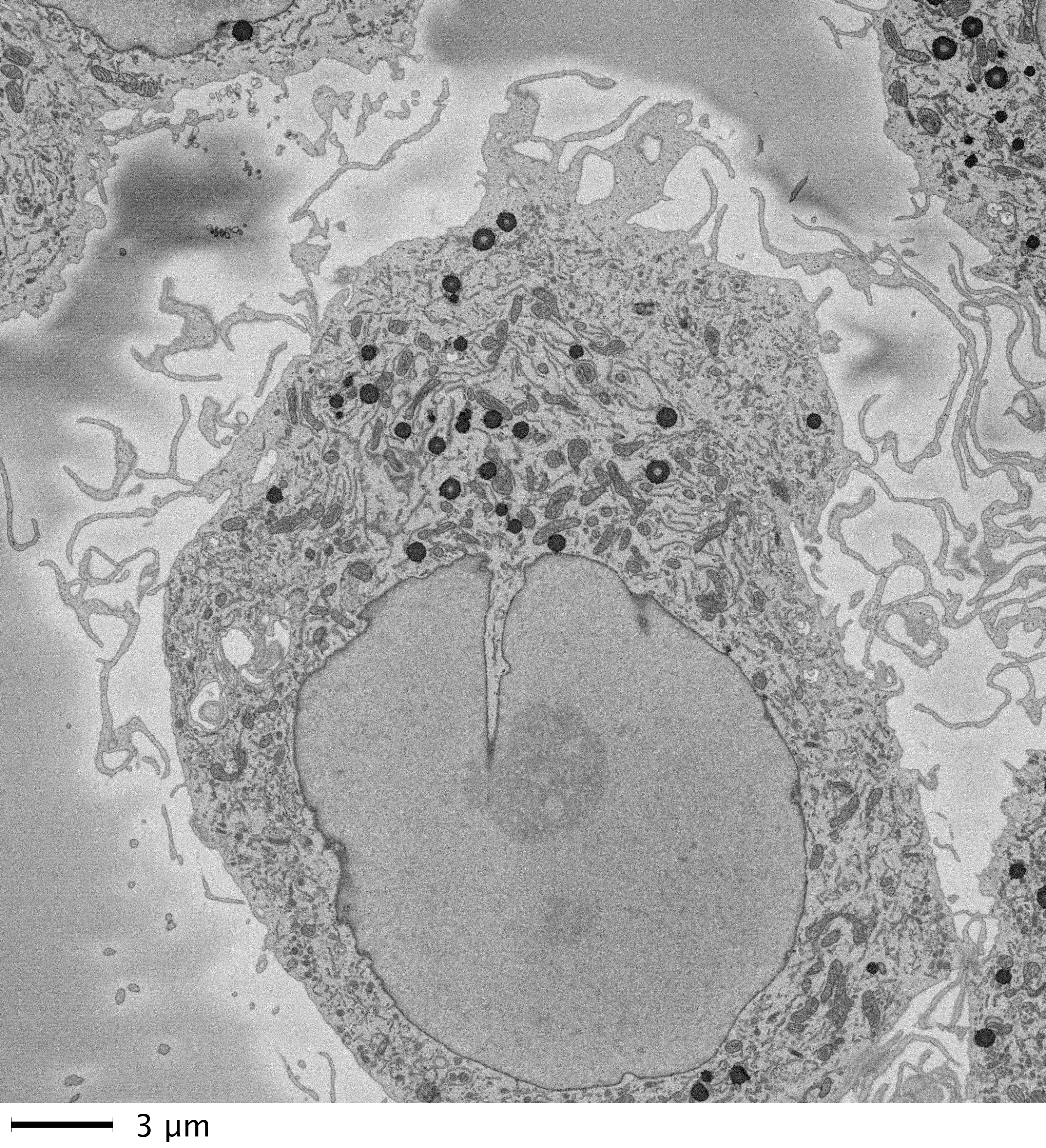

Supplement: Supplementary file 11 — Source Data for Expanded View and Appendix [file 44318_2025_423_MOESM11_ESM.zip › Figure_S1/S1A/Macrophage_SBF-SEM_XY.tif]

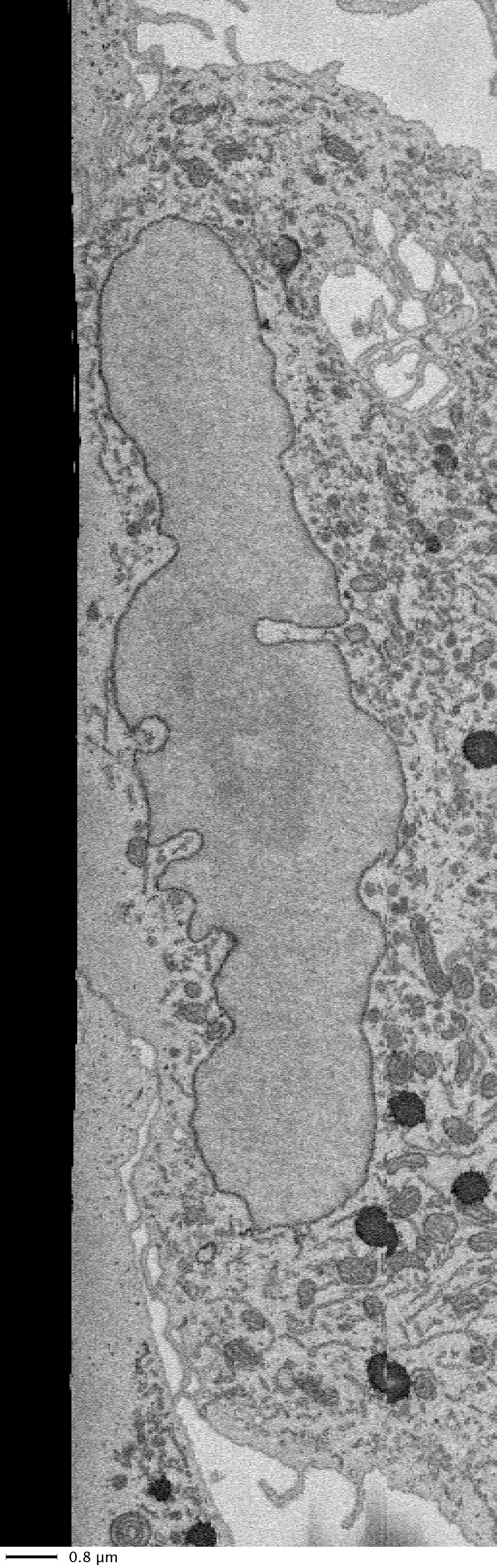

Supplement: Supplementary file 11 — Source Data for Expanded View and Appendix [file 44318_2025_423_MOESM11_ESM.zip › Figure_S1/S1A/Macrophage_SBF-SEM_ZX.tif]

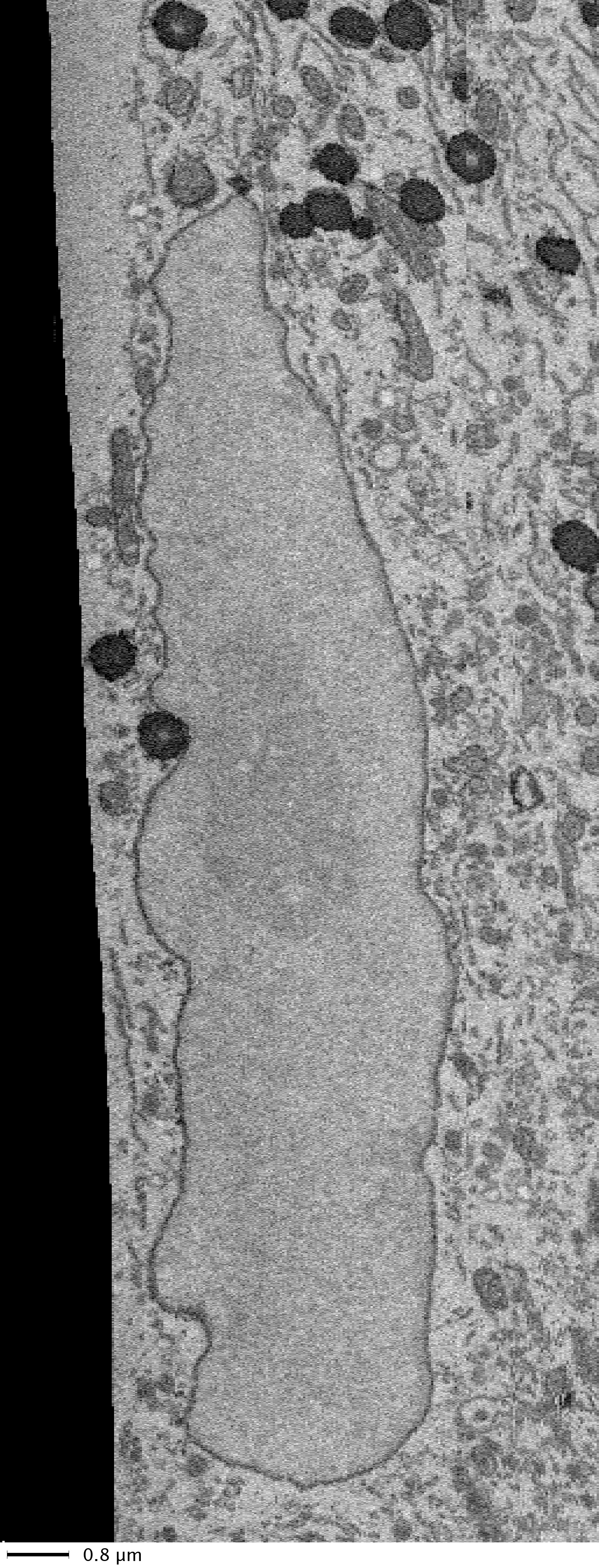

Supplement: Supplementary file 11 — Source Data for Expanded View and Appendix [file 44318_2025_423_MOESM11_ESM.zip › Figure_S1/S1A/Macrophage_SBF-SEM_ZY.tif]
