# Supplementary material for: Nuclear envelope-associated lipid droplets are enriched in cholesteryl esters and increase during inflammatory signaling
Source: EMBO J. 2025 Apr 7;44(10):2774–802. doi: 10.1038/s44318-025-00423-2 (PMC12084420; doi:10.1038/s44318-025-00423-2)
Supplement: Supplementary file 12 — Expanded View Figures [file 44318_2025_423_MOESM12_ESM.pdf]

Expanded View Figures

Figure EV1. SRS reference spectra and whole-cell signal decomposition in A431 cells.

(**A**) Exemplary SRS images at 2860 cm^{-1} Raman shift with exemplary ROIs for defining reference spectra for cellular background, TAG, CE, and water background. (**B**) Reference spectra for the components defined in (**A**): two large areas for cellular background (whose spectra closely overlap), three individual LDs each for TAG and CE, and two large areas for water background (with also closely overlapping spectra). The final TAG and CE reference spectra (bottom row) are generated by subtracting the cellular background spectrum from the TAG and CE spectra measured from LDs (top row). (**C**) SRS lipid intensities (weight coefficients) for the ROIs indicated in (**A**), mean and standard error of pixel-based weights in each of the ROIs. (**D**, **E**) Exemplary false color visualization of the SRS signal decomposition of a 2 h chol loaded A431 cell. TAG and CE visualization for both LDs only (LDs) and whole cell (Cell). (**F**) Comparison of whole-cell lipid amounts measured by SRS and TLC at 2 h chol load (mean per cell ± SEM). Estimated SRS and TLC values are normalized similarly as in Fig. 1H. Number of pixels in (**C**) range from 4 to 18747 (exact numbers in source data). 290 cells in SRS and two technical replicates for TLC in (**F**). Scale bars: 5 µm (A—cellular background, TAG, CE), 20 µm (A—water background), 10 µm (**D**). Source data are available online for this figure.

© The Author(s)

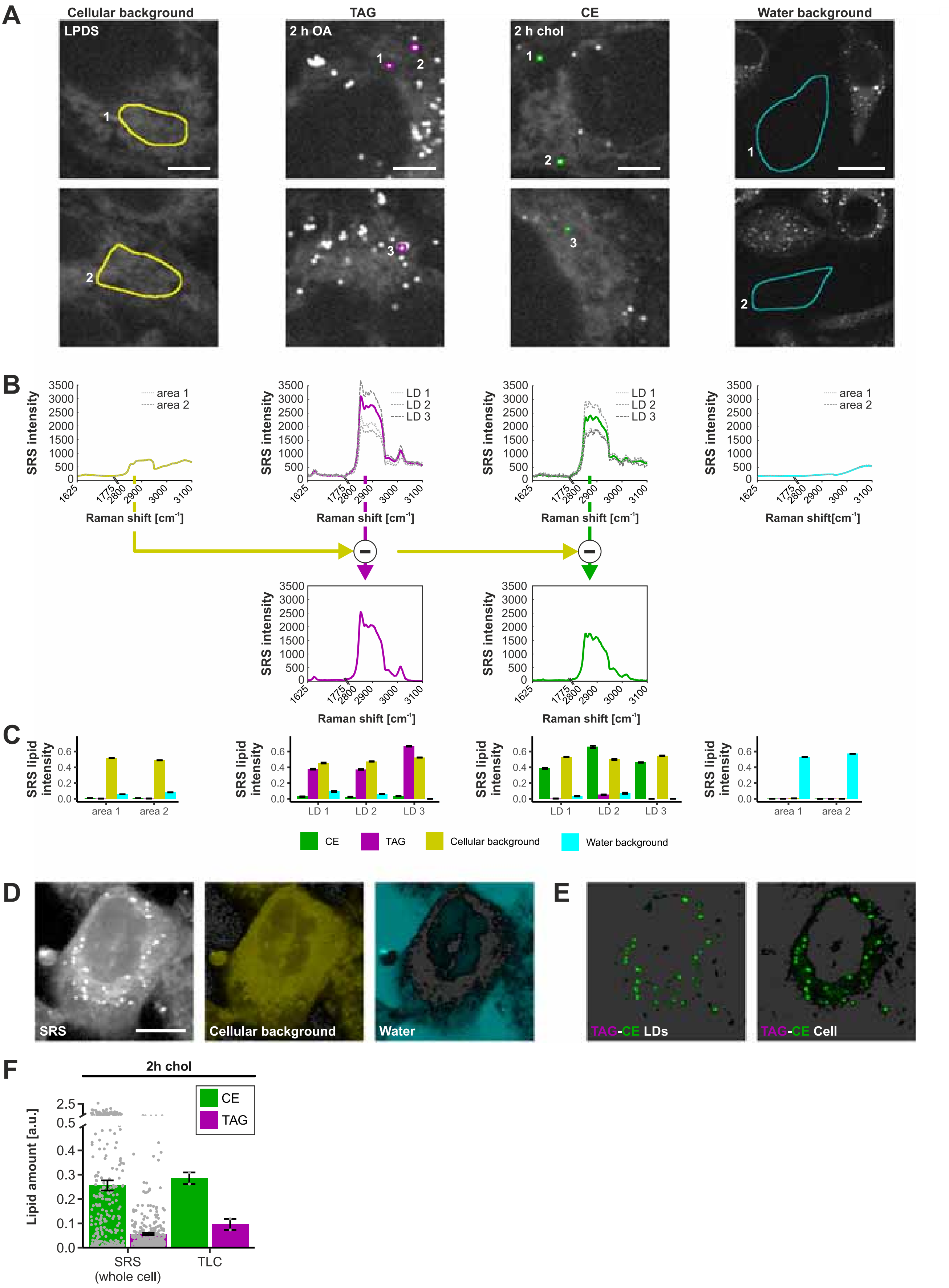
A
Cellular background
LPDS
TAG
2 h OA
CE
2 h chol
Water background
B
SRS intensity
Raman shift [cm-1]
area 1
area 2
LD 1
LD 2
LD 3
C
SRS lipid intensity
CE
TAG
Cellular background
Water background
D
SRS
Cellular background
Water
E
TAG-CE LDs
TAG-CE Cell
F
2h chol
Lipid amount [a.u.]
SRS (whole cell)
TLC

 © The Author(s)

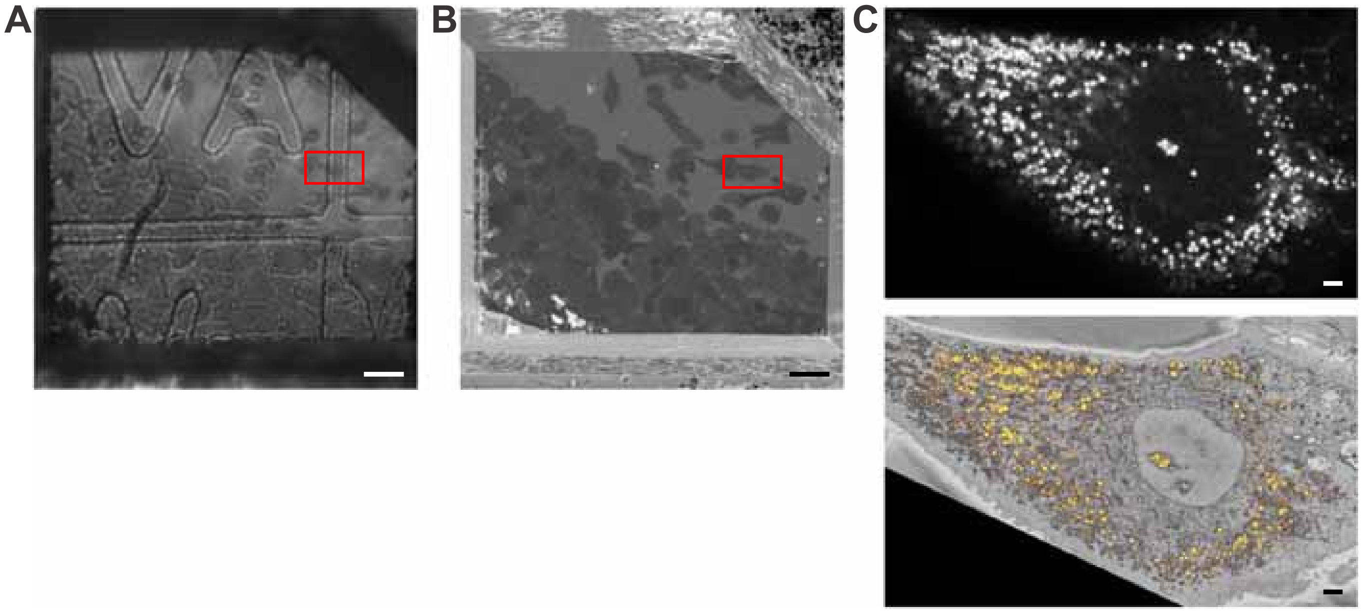

Figure EV2. CLvEM workflow of human primary macrophages.

(**A**) LM image of a block face with embedded human primary macrophages loaded with chol and OA for 2 h and prepared for CLvEM. Lines and labels on the top of the block are used to find the cell of interest for the correlative workflow. The red rectangle indicates the location of the cell of interest. (**B**) SEM image of the block face taken at 15 kV showing the location of cells. (**C**) A confocal LM image showing LDs stained with LD540 and an overlay image showing the correlation between LM and SBF-SEM datasets. The SBF-SEM signal is visualized in grayscale and LM signal in yellow. Scale bars: 80 μm (**A**, **B**), 2 μm (**C**).

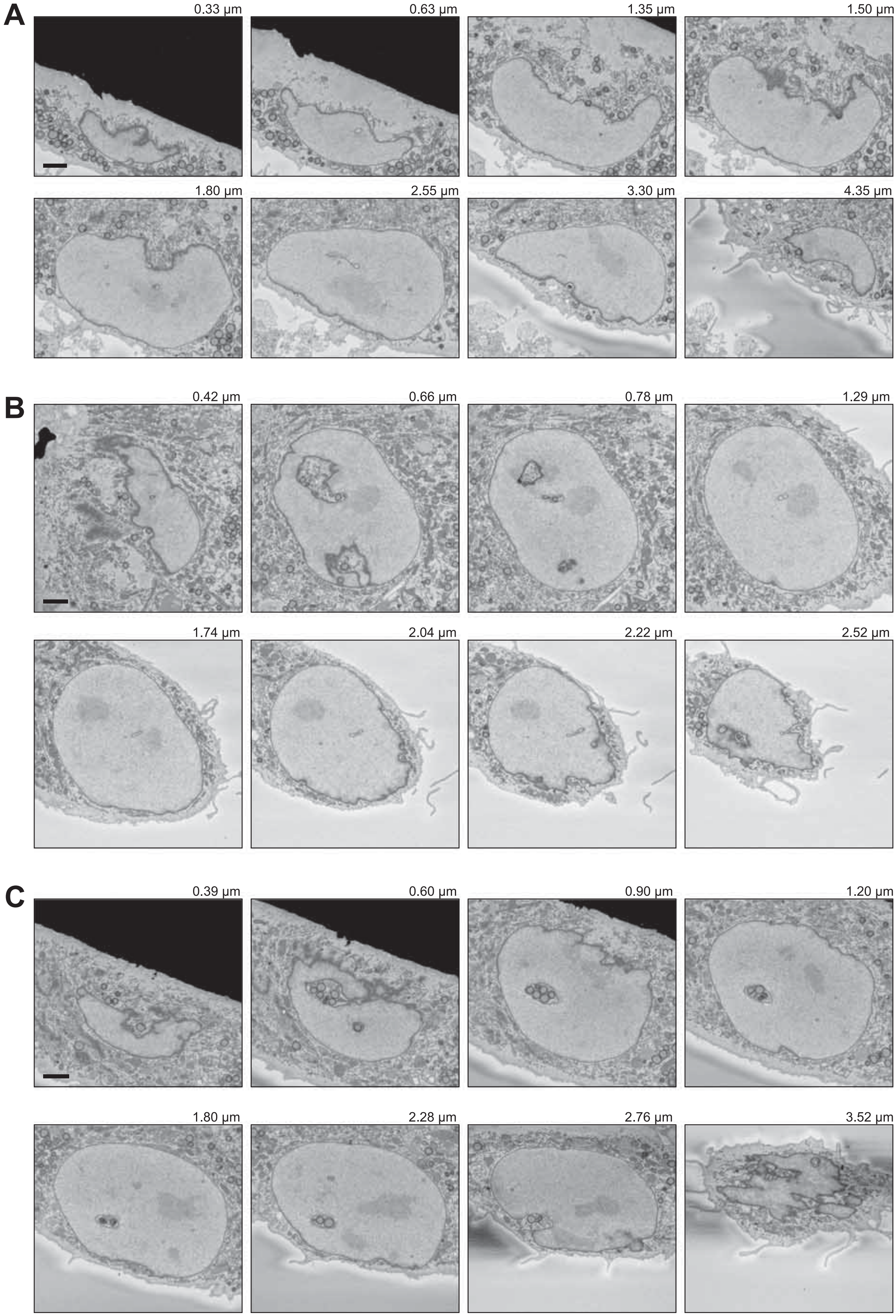
A
0.33 µm
0.63 µm
1.35 µm
1.50 µm
1.80 µm
2.55 µm
3.30 µm
4.35 µm
B
0.42 µm
0.66 µm
0.78 µm
1.29 µm
1.74 µm
2.04 µm
2.22 µm
2.52 µm
C
0.39 µm
0.60 µm
0.90 µm
1.20 µm
1.80 µm
2.28 µm
2.76 µm
3.52 µm

 © The Author(s)

Figure EV3. Volume-EM images of human primary macrophages.

(**A–C**) Cross-sections from SBF-SEM imaging of three individual human primary macrophages, loaded with chol and OA for 2 h, covering the nuclear part of the cells. Cells were imaged using CLvEM and correspond to the images shown in Fig. 4. Numbers above the tiles indicate the distance from the bottom of the corresponding nucleus. Scale bar: 2 µm. Source data are available online for this figure.

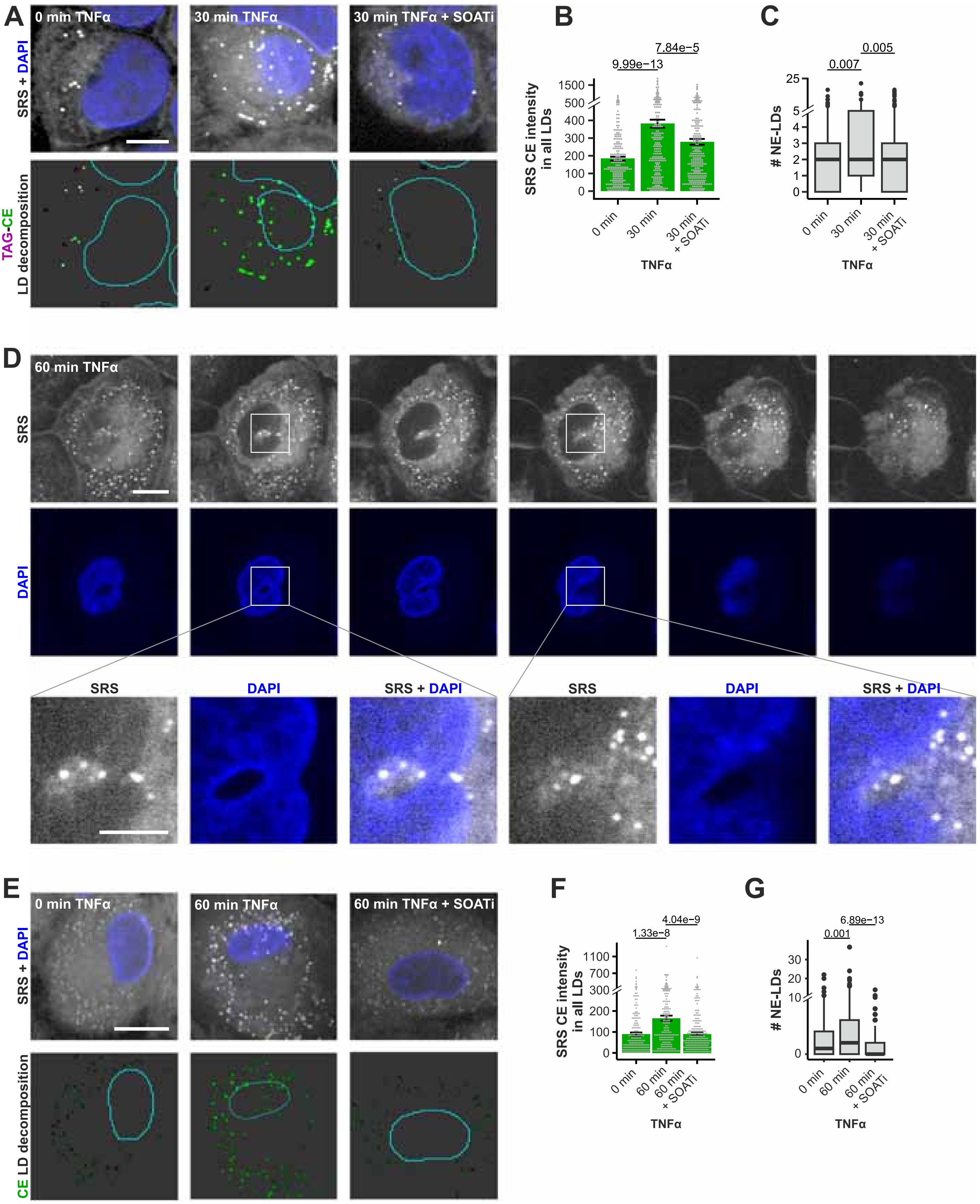
A
0 min TNFα
30 min TNFα
30 min TNFα + SOATi
SRS + DAPI
TAG-CE
LD decomposition
B
SRS CE intensity in all LDs
9.99e−13
7.84e−5
1500
500
400
300
200
100
0
0 min
30 min
30 min + SOATi
TNFα
C
NE-LDs
0.007
0.005
25
5
4
3
2
1
0
0 min
30 min
30 min + SOATi
TNFα
D
60 min TNFα
SRS
DAPI
SRS
DAPI
SRS + DAPI
SRS
DAPI
SRS + DAPI
E
0 min TNFα
60 min TNFα
60 min TNFα + SOATi
SRS + DAPI
CE LD decomposition
F
SRS CE intensity in all LDs
1.33e−8
4.04e−9
1100
700
300
200
100
0
0 min
60 min
60 min + SOATi
TNFα
G
NE-LDs
0.001
6.89e−13
30
20
10
0
0 min
60 min
60 min + SOATi
TNFα

 © The Author(s)

Figure EV4. TNFα-induced NE-LDs and their SOAT sensitivity.

(**A**) Exemplary SRS images and signal decompositions of A431 cells at 0 and 30 min TNFα treatment ± SOAT inhibition. (**B**) Quantification of SRS CE intensity in LDs upon treatments as in (**A**), data presented as mean ± SEM per cell. *p* values from *t*-tests. (**C**) Number of NE-LDs upon treatments as in (**A**), *p* values from Mann–Whitney *U*-tests, boxplots according to ggplot (see Methods). (**D**) An exemplary z-stack of SRS and DAPI signals at 60 min TNFα treatment showing an NE tunnel containing LDs in a human primary macrophage. (**E**) Exemplary SRS images and signal decompositions of human primary macrophages at 0 and 60 min TNFα treatment ± SOAT inhibition. (**F**) Quantification of SRS CE intensity in LDs upon treatments as in (**E**), data presented as mean ± SEM per cell. *p* values from *t*-tests. (**G**) Number of NE-LDs upon treatments as in (**E**), *p* values from Mann–Whitney *U*-tests, boxplots according to ggplot (see Methods). Cell numbers: 228, 270, 315 for 0, 30, 30 min TNFα + SOATi in (**B**, **C**); 225, 210, 278 for 0, 60, 60 min TNFα + SOATi in (**F**, **G**). Scale bars: 10 µm (**A**, **D**, **E**) and 5 µm (**D** inset), distance of consecutive z-slices 0.75 µm. Source data are available online for this figure.

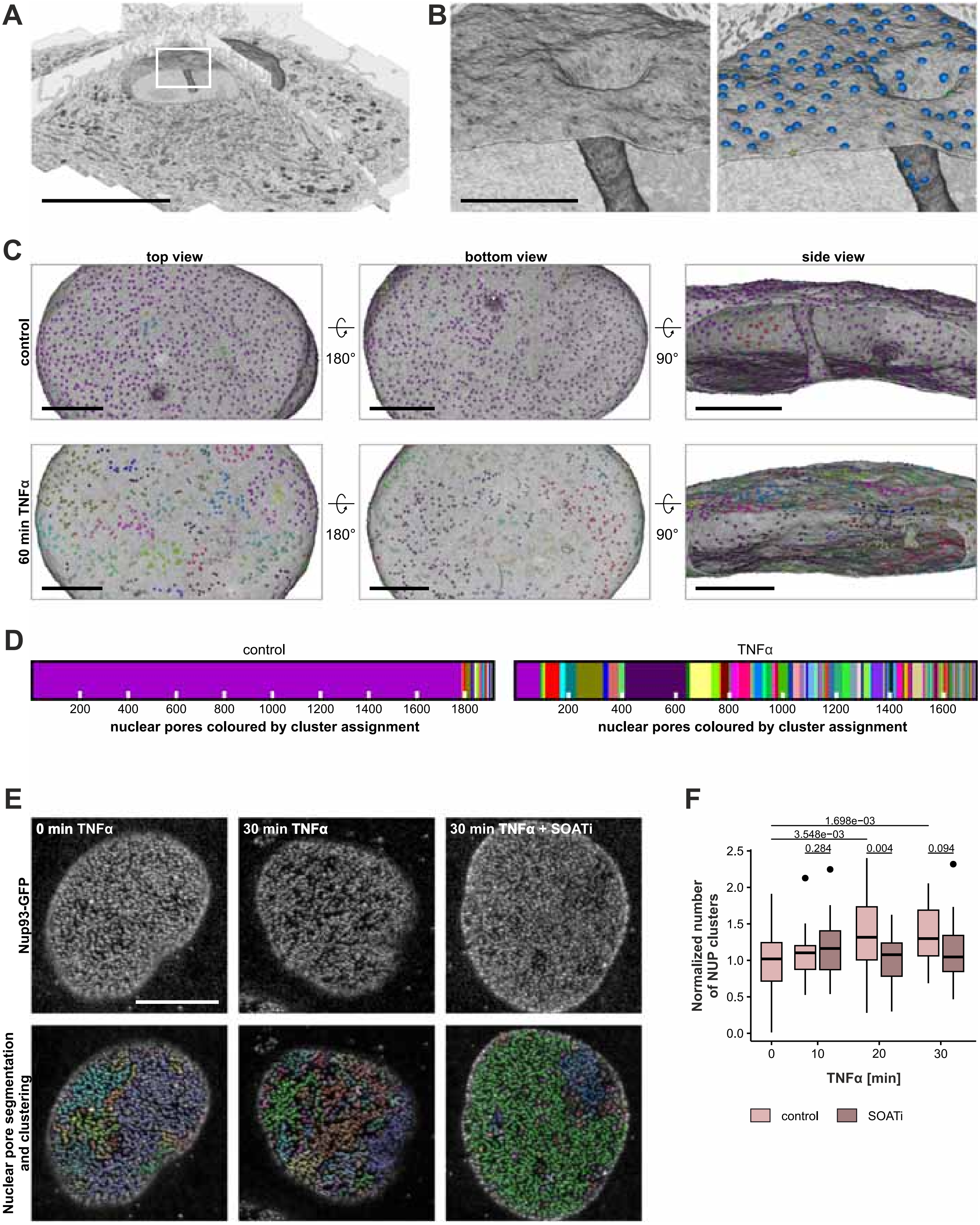
A
B
C
top view
bottom view
side view
control
180°
90°
60 min TNFα
180°
90°
D
control
TNFα
200 400 600 800 1000 1200 1400 1600 1800
nuclear pores coloured by cluster assignment
200 400 600 800 1000 1200 1400 1600
nuclear pores coloured by cluster assignment
E
0 min TNFα
30 min TNFα
30 min TNFα + SOATi
Nup93-GFP
Nuclear pore segmentation and clustering
F
1.698e−03
3.548e−03
0.284
0.004
0.094
Normalized number of NUP clusters
2.5
2.0
1.5
1.0
0.5
0.0
0 10 20 30
TNFα [min]
control
SOATi

Figure EV5. Clustering of nuclear pores in TNFα-treated cells.

(**A**) 3D visualization of the whole control primary human macrophage cell imaged using FIB-SEM. The image combines orthoslices showing cross-sections from different planes and the volume rendering of the nuclear envelope. (**B**) Insets from (**A**) showing the magnified view of the nuclear envelope (left) and the model of the detected nuclear pores (right). (**C**) Analysis of spatial clustering of the nuclear pores in control and 1 h TNFα-treated macrophages. Pores belonging to the same cluster are visualized with the same color on the top of the 3D volume rendering of the nuclear envelope. The images show the bottom and the side views of the nuclei with the model of the detected nuclear pores. The NEs are clipped at the side views to expose the internal part of the nucleus. (**D**) Visualization of the number and size of nuclear pore clusters. Each colored stripe indicates one cluster, and its width shows the number of nuclear pores belonging to the cluster. (**E**) Images of Nup93-GFP expressing A431 cells treated with TNFα ± SOATi as indicated. The lower row shows the segmentation of nuclear pores and contours colored by cluster assignment. (**F**) Number of clusters normalized to 0 min treatment in A431 cells under 0, 10, 20, and 30 min of TNFα treatment ± SOATi. Cell numbers: 37, 31, 27, 38, 30, 28, and 24 from left to right, data pooled from two biological replicates. *p* values from *t*-tests and boxplots according to ggplot (see Methods). Scale bars: 10 µm (**A**, **E**), 2 µm (**B**), 3 µm (**C**). Source data are available online for this figure.

© The Author(s)
